# ACONITASE 3 is part of theANAC017 transcription factor-dependent mitochondrial dysfunction response

**DOI:** 10.1093/plphys/kiab225

**Published:** 2021-05-12

**Authors:** Jesús Pascual, Moona Rahikainen, Martina Angeleri, Sara Alegre, Richard Gossens, Alexey Shapiguzov, Arttu Heinonen, Andrea Trotta, Guido Durian, Zsófia Winter, Jari Sinkkonen, Jaakko Kangasjärvi, James Whelan, Saijaliisa Kangasjärvi

**Affiliations:** 1 Department of Life Technologies, Molecular Plant Biology, University of Turku, Turku FI-20014, Finland; 2 Faculty of Biological and Environmental Sciences, Organismal and Evolutionary Biology Research Programme, University of Helsinki, Helsinki FI-00014, Finland; 3 Viikki Plant Science Center, University of Helsinki, Helsinki FI-00014, Finland; 4 Institute of Plant Physiology, Russian Academy of Sciences, Moscow 127276, Russia; 5 Turku Centre for Biotechnology, University of Turku and Åbo Akademi University, Turku FI-20520, Finland; 6 Institute of Biosciences and Bioresources, National Research Council of Italy, Sesto Fiorentino 50019, Italy; 7 Department of Chemistry, Instrument Centre, University of Turku, Turku FI-20014, Finland; 8 Department of Animal, Plant and Soil Science, ARC Centre of Excellence in Plant Energy Biology, La Trobe University, Bundoora 3086, Australia; 9 Department of Agricultural Sciences, Faculty of Agriculture and Forestry, University of Helsinki, Helsinki FI-00014, Finland

## Abstract

Mitochondria are tightly embedded within metabolic and regulatory networks that optimize plant performance in response to environmental challenges. The best-known mitochondrial retrograde signaling pathway involves stress-induced activation of the transcription factor NAC DOMAIN CONTAINING PROTEIN 17 (ANAC017), which initiates protective responses to stress-induced mitochondrial dysfunction in Arabidopsis (*Arabidopsis thaliana*). Posttranslational control of the elicited responses, however, remains poorly understood. Previous studies linked protein phosphatase 2A subunit PP2A-B’γ, a key negative regulator of stress responses, with reversible phosphorylation of ACONITASE 3 (ACO3). Here we report on ACO3 and its phosphorylation at Ser91 as key components of stress regulation that are induced by mitochondrial dysfunction. Targeted mass spectrometry-based proteomics revealed that the abundance and phosphorylation of ACO3 increased under stress, which required signaling through ANAC017. Phosphomimetic mutation at *ACO3-Ser91* and accumulation of ACO3^S91D^-YFP promoted the expression of genes related to mitochondrial dysfunction. Furthermore, ACO3 contributed to plant tolerance against ultraviolet B (UV-B) or antimycin A-induced mitochondrial dysfunction. These findings demonstrate that ACO3 is both a target and mediator of mitochondrial dysfunction signaling, and critical for achieving stress tolerance in Arabidopsis leaves.

## Introduction

Plants respond to environmental stimuli by metabolic adjustments that enable growth and survival when biotic and abiotic challenges occur. In mitochondria, stress-induced disruption of metabolic reactions can cause accumulation of metabolic intermediates and reactive oxygen species (ROS), which serve well-documented signaling functions in stress-exposed tissues (Obata and Fernie, 2012; Foyer et al., 2017; Waszczak et al., 2018). Dysfunctional mitochondria communicate through retrograde signaling pathways, which alter the expression of nuclear genes with consequent readjustments in cellular redox balance, metabolism, and detoxification (Ho et al., 2008; Van Aken and Whelan, 2012; De Clercq et al., 2013; Ng et al., 2013). Collectively, the protective response is termed the mitochondrial dysfunction response (MDR). An increasing body of evidence indicates that mitochondrial dysfunction is integral to various stress responses in plant cells (Meng et al., 2019; Rasool et al., 2020). MDR can, therefore, be elicited by environmental, pharmacological, and genetic perturbations, such as plant exposure to the mitochondrial complex III inhibitor antimycin A (AA) or UV-B irradiation, which trigger partially overlapping transcriptional responses (Umbach et al., 2012; Willems et al., 2016).

Signaling from dysfunctional mitochondria deploy the transcription factors NAC DOMAIN CONTAINING PROTEIN 13 (ANAC013) and ANAC017, which undergo stress-induced translocation from the endoplasmic reticulum (ER) to the nucleus (De Clercq et al., 2013; Ng et al., 2013). In the nucleus, the signaling functions of ANAC013 and ANAC017 are inhibited by RADICAL-INDUCED CELL DEATH 1 (RCD1), which coordinates stress adaptation in response to mitochondrial and chloroplastic ROS signals (Shapiguzov et al., 2019). While the mechanisms underlying transcriptional regulation of the MDR have started to emerge, understanding how the protective response is posttranslationally controlled by adjustments in enzymatic activities, protein interactions, protein turnover, or localization of proteins is still limited.

Among metabolic enzymes, aconitases (ACOs; EC 4.2.1.3) catalyze the conversion of citrate to isocitrate and connect with various pathways of basic metabolism. In mitochondria, the ACO-catalyzed reaction forms part of the tricarboxylic acid (TCA) cycle, while cytosolic ACOs contribute to the glyoxylate cycle, glutamate and glutamine biosynthesis, and ammonium fixation, and hence influence both carbon and nitrogen (N_2_) metabolism (Mhamdi et al., 2010; Hooks et al., 2014). In Arabidopsis, ACO is present as three isoforms, ACO1–3. Three-day-old Arabidopsis seedlings displayed strong expression of ACO3, which was predominantly cytosolic and acted in lipid mobilization (Hooks et al., 2014). In 4-week-old plants, however, ACO3 was predominantly detected in a cell fraction enriched in mitochondria (Bernard et al., 2009). The regulatory mechanism behind such dual ACO3 localization remains unknown.

ROS can directly affect ACO function, as the ACO-driven reaction of the TCA cycle was reported sensitive to hydrogen peroxide (H_2_O_2_; Verniquet et al., 1991). In tobacco (*Nicotiana tabacum*), accumulation of the ACO substrate, citrate, was coupled with increased capacity of alternative oxidases (AOXs), which form a safety valve against over-reduction of the mitochondrial electron transport chain (Vanlerberghe and Mclntosh, 1996; Gray et al., 2004; Gupta et al., 2012; Selinski et al., 2018). In the cytoplasm, ACO can also have moonlighting functions that affect cellular redox balance via altered antioxidant activities; for example, Arabidopsis ACO3 was proposed to regulate the stability of *CHLOROPLAST SUPEROXIDE DISMUTASE 2* mRNA (Moeder et al., 2007). Together, these findings suggest that alterations in ACO function can influence metabolic interactions, redox homeostasis, and signaling in stress-exposed plants.

Arabidopsis ACO3 carries a unique phosphorylation site at Ser91, which is not present in ACO1 or ACO2 (Konert et al., 2015). Furthermore, ACO3 interacted with protein phosphatase 2A regulatory subunit B’γ (PP2A-B’γ) in the cytosol, and a knock-down *pp2a-b’γ* mutant displayed increased phosphorylation of Ser91 on ACO3, suggesting that ACO3 is posttranslationally controlled by PP2A-B’γ (Konert et al., 2015). PP2A-B’γ is a signaling component that controls organellar ROS signaling, salicylic acid signaling, stress-induced metabolic changes, and the abundance of the specific AOX isoforms 1A and 1D in leaf mitochondria (Trotta et al., 2011; Li et al., 2014; Konert et al., 2015; Durian et al., 2016; Rahikainen et al., 2017; Durian et al., 2020; Rasool et al., 2020). However, the physiological significance of ACO3 phosphorylation, and its possible connection to AOX function, has remained elusive.

Here we report that ACO3 and its phosphorylation at Ser91 contribute to plant tolerance against UV-B or AA-induced stress, and that stress-induced increase in the abundance and phosphorylation of ACO3 requires signaling through ANAC017. Altogether, we suggest that ACO3 is a component of the MDR in Arabidopsis.

## Results

### ACO3 phosphomutant lines do not display visual phenotypes in the absence of stress

**Figure 1:**
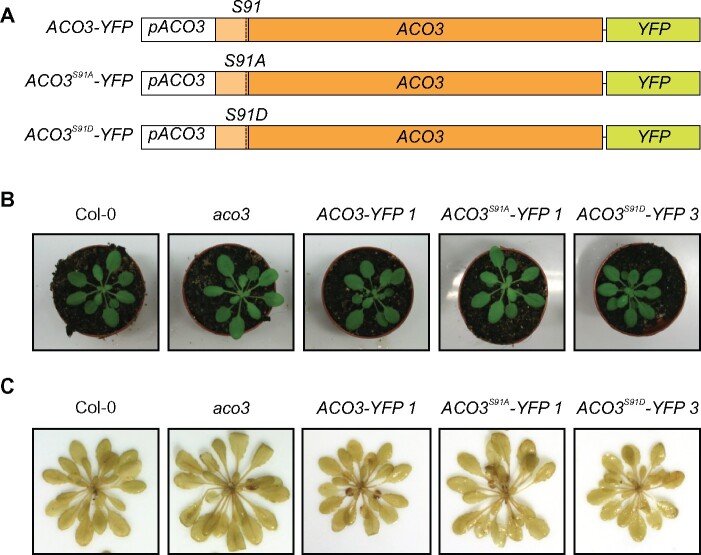
Overview of the *ACO3* WT and Ser91-mutated complementation lines. A, Schematic representation of the gene constructs used to complement *aco3.* Constructs were expressed under the control of the native *pACO3* promoter. The mitochondrial targeting sequence is located at the 5′ end of the protein-coding sequence (pale orange) and separated with a dashed line. The codon corresponding to the phosphorylation site Ser91 and the introduced amino acid substitutions are indicated. B, Phenotypes of WT (Col-0), *aco3, aco3* expressing *pACO3::ACO3-YFP* (*ACO3-YFP 1*)*, pACO3::ACO3^S91A^-YFP* (*ACO3^S91A^-YFP 1*) or *pACO3::ACO3^S91D^-YFP* (*ACO3^S91D^-YFP 3*) grown in short-day conditions under 120 μmol photons m^−2^ s^−1^. C, Generation of H_2_O_2_ in WT (Col-0), *aco3, aco3* expressing *pACO3::ACO3-YFP* (*ACO3-YFP 1*)*, pACO3::ACO3^S91A^-YFP* (*ACO3^S91A^-YFP 1*), or *pACO3::ACO3^S91D^-YFP* (*ACO3^S91D^-YFP 3*) under growth light. H_2_O_2_ was detected by the formation of brown precipitate in the leaves after staining with DAB.

To investigate the physiological role of ACO3 phosphorylation at Ser91, we complemented Arabidopsis *aco3* knock-out mutant (*aco3*) with the ACO3 coding sequence in which we introduced point mutations to substitute Ser91 with either alanine (*ACO3^S91A^*) or aspartic acid (*ACO3^S91D^*), abrogating the Ser91 phosphosite or mimicking constitutive phosphorylation, respectively. To monitor the subcellular localization of ACO3, yellow fluorescent protein (YFP) was fused to the C-terminus of the *ACO3*, *ACO3^S91A^*, and *ACO3^S91D^* constructs (Figure 1A). Neither the knockout of ACO3 nor the mutations introduced at Ser91 of ACO3 had any effect on the visual phenotypes of 5-week-old plants when grown in the absence of external stress under 120 μmol photons m^−2^ s^−1^ (Figure 1B). Accordingly, diaminobenzidine (DAB) staining showed only very little brown precipitate, indicating minute H_2_O_2_ accumulation in all genotypes (Figure 1C). Hence, alterations in ACO3 phosphorylation did not promote apparent stress responses in standard growth conditions.

### ACO3 localizes to mitochondria in Arabidopsis leaves

**Figure 2:**
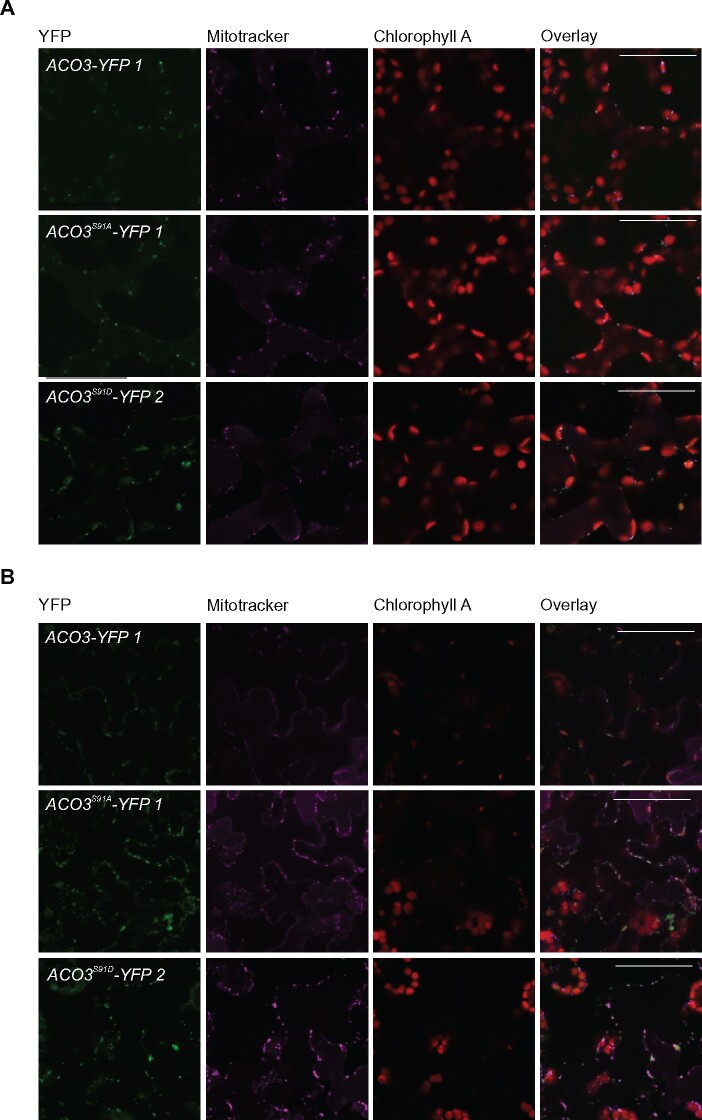
Subcellular localization of YFP-tagged WT and Ser91-mutated ACO3. A, Fluorescence confocal microscope images showing ACO3-YFP localization in leaf epidermal cells of *aco3* lines expressing *pACO3::ACO3-YFP* (*ACO3-YFP 1*)*, pACO3::ACO3^S91A^-YFP* (*ACO3^S91A^-YFP 1*) or *pACO3::ACO3^S91D^-YFP* (*ACO3^S91D^-YFP 2*) in normal growth conditions. B, Localization of ACO3-YFP in leaf epidermal cells of *aco3* lines expressing *pACO3::ACO3-YFP* (*ACO3-YFP 1*), *pACO3::ACO3^S91A^-YFP* (*ACO3^S91A^-YFP 1*), or *pACO3::ACO3^S91D^-YFP* (*ACO3^S91D^-YFP 2*) as detected 24 h after exposure of plants to UV-B stress (1.5 W m^−2^ for 45 min). YFP fluorescence co-localizes with Mitotracker fluorescence in the mitochondria. A and B, Scale bars correspond to 50 μm.

The ACO3 Ser91 resides on the mature ACO3 protein and is located close to the N-terminus of the protein sequence, after the predicted mitochondrial target peptide of 78 amino acids (Konert et al. 2015). The location of the phosphosite close to the target peptide prompted us to investigate whether the phosphorylation of Ser91 affects the subcellular localization of the protein. ACO3-YFP, ACO3^S91A^-YFP, and ACO3^S91D^-YFP were imaged in leaves excised from 4-week-old plants, using Mitotracker to compare the localization of ACO3 variants to the localization pattern of mitochondria (Figure 2A). All three different forms of ACO3 were localized predominantly to mitochondria in leaf epidermal cells (Figure 2A). Similar localization of ACO3 was detected in Arabidopsis leaf mesophyll cells (Supplemental Figure S1) and when ACO3 localization was imaged 24 h after exposing the plants to UV-B irradiation (Figure 2B). These data suggested that phosphorylation at Ser91 does not affect the mitochondrial import of ACO3.

### Phosphomimetic mutation at Ser91 leads to increased abundance of ACO3

**Figure 3:**
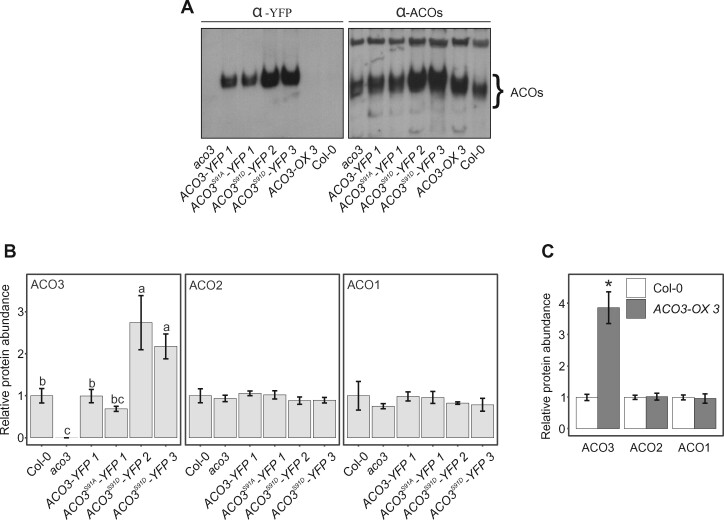
Analysis of ACO complex formation and relative quantification of ACO isoforms in leaves of WT and *ACO3* mutants. A, Complex formation of ACO3-YFP and ACO1-3 in WT (Col-0), *aco3*, and *aco3* complemented by *pACO3::ACO3-YFP* (*ACO3-YFP 1*), *pACO3::ACO3^S91A^-YFP* (*ACO3^S91A^-YFP 1*) or *pACO3::ACO3^S91D^-YFP* (*ACO3^S91D^-YFP 2* and *ACO3^S91D^-YFP 3*) and a *35S::ACO3* over-expressor line (*ACO3-OX 3*). Total foliar proteins were separated on CN-PAGE and YFP and ACOs were detected with α-YFP and α-ACO antibodies by immunobloting. B, Relative abundance of ACO1-3 isoforms in WT (Col-0), *aco3, aco3* expressing *pACO3::ACO3-YFP* (*ACO3-YFP 1*)*, pACO3::ACO3^S91A^-YFP* (*ACO3^S91A^-YFP 1*) or *pACO3::ACO3^S91D^-YFP* (*ACO3^S91D^-YFP 2* and *ACO3^S91D^-YFP 3*) as detected by PRM. Values represent the mean of *n* = 3 ± sd. Letters indicate significant differences (one-way ANOVA with Tukey’s HSD test, *P* < 0.05). C, Relative abundance of ACO1–3 isoforms in WT and *35S::ACO3* over-expression line *ACO3-OX 3*. Error bars represent sd (*n* = 3). Asterisks indicate values significantly different from the WT (Col-0) sample for each protein isoform; Student’s *t* test, *P* < 0.05.

Separation of protein complexes by clear native (CN) gel electrophoresis revealed no differences in the pattern of ACO-containing complexes in the different transgenic *ACO3* mutant lines, suggesting that the YFP fusion did not affect ACO3 function at the level of protein complex formation (Figure 3A). However, increased ACO3 complex abundance, as compared to wild-type (WT) plants, was observed in two independent *ACO3^S91D^-YFP* lines, *ACO3^S91D^-YFP 2*, and *ACO3^S91D^-YFP 3*, and in a 35S::ACO3 over-expressor line *ACO3-OX 3* (Figure 3A).

To analyze the relative abundance of ACO1-3 isoforms, we developed a targeted Parallel Reaction Monitoring (PRM) method for their label-free quantification by mass spectrometry (MS). This MS-based approach revealed a more than two-fold increase in the abundance of ACO3^S91D^-YFP in *ACO3^S91D^-YFP* lines, as compared to the abundance of ACO3 in WT (Figure 3B;Supplemental Figure S2A). In two independent experiments, the *ACO3^S91D^-YFP 2* line showed 2.7- (Figure 3B) and 6.0-fold (Supplemental Figure S2A) increases in the abundance of ACO3^S91D^-YFP, while a 2.2-fold increase in ACO3^S91D^-YFP abundance was recorded in *ACO3^S91D^-YFP 3* (Figure 3B). In contrast, the abundance of ACO3^S91A^-YFP in *ACO3^S91A^-YFP 1* (Figure 3B) or *ACO3^S91A^-YFP 2* (Supplemental Figure S2A) did not significantly differ from that of ACO3 in WT. The abundance of ACO3-YFP in *ACO-YFP 1* (Figure 3B) and *ACO-YFP 3* (Supplemental Figure S2A) was similar to the abundance of ACO3 in the WT.

The levels of ACO1 and ACO2 did not significantly differ between the WT, *aco3*, or the transgenic *ACO-YFP*, *ACO3^S91A^-YFP*, and *ACO3^S91D^-YFP* lines, suggesting that the abundance of ACO1 or ACO2 was not affected by changes in the abundance of ACO3 (Figure 3B; for data on second independent transgenic lines, see Supplemental Figure S2A). Likewise, the *ACO3-OX 3* and *ACO3-OX 8* lines, which showed 3.9- and 4.5-fold increased ACO3 abundance, respectively, contained WT levels of ACO2 and ACO1 (Figure 3C;Supplemental Figure S2A). The observed increase in the total ACO abundance in the *ACO3^S91D^-YFP* lines (Figure 3A) could therefore be attributed to the increased abundance of ACO3^S91D^-YFP. Hence, the phosphomutation of ACO3 at Ser91 promoted increased ACO3 abundance, and this had an impact on the total ACO abundance in the nonstressed plants.

### The abundance of ACO1-3 isoforms increases in an ANAC017-dependent manner in response to stress

**Figure 4:**
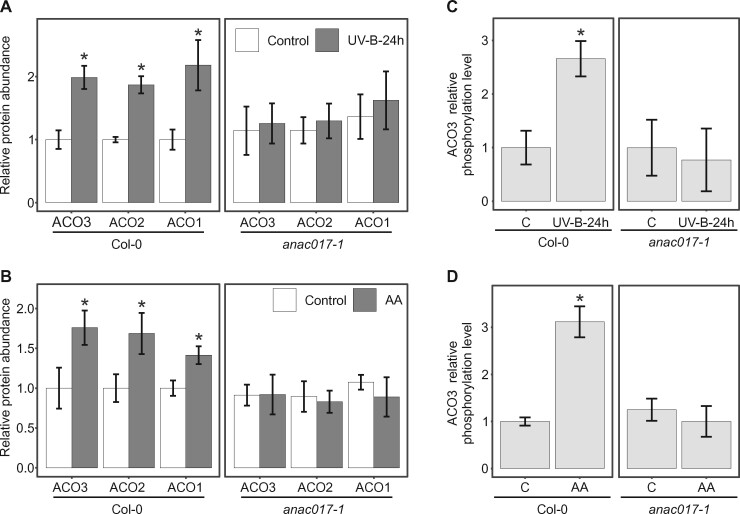
ACO3as a component of the MDR. A, Relative abundance of ACO 1–3 in WT (Col-0) and *anac017-1* mutant plants in control conditions and 24 h after UV-B treatment (1.5 W m^−2^, 45 min) as detected by PRM. B, Relative abundance of ACO1–3 in WT (Col-0) and *anac017-1* plants in control conditions and 10 h after treatment with 50 μM AA, as detected by PRM. C, tSIM/PRM relative quantification of Ser91 phosphorylation of ACO3 in WT (Col-0) and *anac017-1* in control conditions and 24 h after UV-B treatment (1.5 W m^−2^, 45 min). D, tSIM/PRM relative quantification of Ser91 phosphorylation of ACO3 in WT (Col-0) and *anac017-1* in control conditions and 10 h after treatment with 50 μM AA. A–D, Error bars represent sd (*n* * *= 3). Asterisks indicate values significantly different from the control sample for each genotype; Student’s *t* test, *P* < 0.05.

Next, we explored whether the abundance of ACO1-3 isoforms is modulated in response to UV-B or AA-induced mitochondrial dysfunction. PRM quantification showed that the abundance of all the ACO1-3 isoforms was elevated in WT leaf tissues harvested 24 h after a harsh UV-B treatment (1.5 W m^−2^, 45 min; Figure 4A). Similar increases in the abundance of ACO1-3 isoforms were found 10 h after AA treatment (Figure 4B). In contrast, the abundance of RUBISCO LARGE SUBUNIT (RBCL; ATCG00490), RUBISCO SMALL SUBUNIT 1A (RBCS1A; AT1G67090), or GLYCERALDEHYDE 3-PHOSPHATE DEHYDROGENASE A SUBUNIT (GAPA; AT3G26650) did not show any significant changes in response to either of the stress treatments (Supplemental Figure S3, A and B). Parallel analysis of *anac017-1* knock-out mutant showed that UV-B-induced accumulation of ACO1-3 isoforms was abolished in the absence of ANAC017, the master transcriptional regulator of the MDR (Figure 4A). Similarly, an AA-induced increase in the abundance of ACO1-3 isoforms required functional ANAC017 (Figure 4B). Furthermore, the abundance of ACO isoforms was also increased in *rcd1*, which is deficient in a negative regulator of MDR and shows constitutive accumulation of MDR markers, including AOXs and SULFOTRANSFERASE 12 (SOT12; Supplemental Figure S4A; Shapiguzov et al., 2019).

### The level of ACO3 phosphorylation at Ser91 increases in response to stress

A method combining targeted Selected Ion Monitoring with PRM (tSIM/PRM) was next developed to improve the sensitivity, selectivity, and accuracy in the quantification of ACO3 phosphorylation at Ser91. This methodological approach revealed that the stoichiometry of ACO3-Ser91 phosphorylation increased in both UV-B- and AA-treated WT, as compared to plants grown under control conditions (Figure 4, C and D). On Phos-tag gels, bands corresponding to ACO3 Ser91 phosphorylation could not be detected when transgenic ACO3-YFP lines were analyzed with the immunoblot approach (Supplemental Figure S5). The *anac017-1* mutant did not display these stress-induced changes in its levels of ACO3 phosphorylation (Figure 4, C and D). Therefore, both the abundance and phosphorylation of ACO3 increased in an ANAC017-dependent manner in response to external stress signals. However, stress-induced activation of MAP kinases (MPKs) 3 and 6 was evident in *anac017-1*, as demonstrated by increased total abundance and phosphorylation of the regulatory residues within the active centers of these stress-responsive protein kinases upon exposure to UV-B or AA (Supplemental Figure S4, B and C). These findings suggested that the *anac017-1* mutant did not suffer from a general incapability to trigger stress responses.

**Figure 5:**
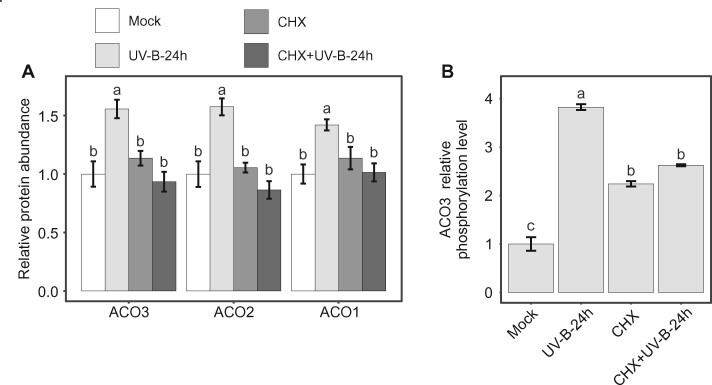
Effect of inhibition of de novo protein synthesis on abundance of ACO isoforms and ACO3 phosphorylation. A, Relative abundance of ACO1–3 isoforms in WT (Col-0) in control conditions (Mock), 24 h after treatment with UV-B (1.5 W m^−2^, 45 min), after treatment with 25 µM CHX, and after CHX treatment followed by UV-B treatment, as detected by PRM. Error bars represent sd (*n* = 3). Letters indicate significant differences (one-way ANOVA with Tukey’s HSD test, *P* < 0.05). B, tSIM/PRM quantification of ACO3 Ser91 phosphorylation in WT (Col-0) plants in control conditions (Mock), 24 h after treatment with UV-B (1.5 W m^−2^, 45 min), after treatment with 25 µM CHX, and after CHX treatment followed by UV-B treatment. The values are presented relative to WT + mock. Values represent the mean of *n* = 3 ± sd. Letters indicate significant differences (one-way ANOVA with Tukey’s HSD test, *P* < 0.05).

To test if stress-induced increase in the level of ACO3-Ser91 phosphorylation required *de novo* protein synthesis, we quantified the abundance of ACO1-3 and the level of ACO3-Ser91 phosphorylation in plants treated with the protein synthesis inhibitor cycloheximide (CHX). The CHX treatment did not induce changes in ACO1-3 abundance in control conditions, but prevented their stress-induced accumulation in response to UV-B irradiation (Figure 5A). The abundance of RBCL, RBCS1A, and GAPA did not show any significant changes in any of the conditions studied (Supplemental Figure S3C). Hence, stress-induced increase in ACO1-3 abundance required *de novo* synthesis of the protein. It was therefore notable that treatment with CHX alone was sufficient to trigger a 2.2-fold increase in the relative level of ACO3-Ser91 phosphorylation, as compared to the level of ACO3 phosphorylation in the control samples (Figure 5B). However, the level of ACO3 phosphorylation did not further increase when the CHX-treated plants were exposed to UV-B, and did not reach the level of phosphorylation detected in plants exposed only to UV-B stress (Figure 5B). These findings suggested that stress-induced phosphorylation can occur on pre-existing ACO3, but reaching the full level of ACO3 phosphorylation may require *de novo* synthesis of the protein.

### Total foliar ACO activity is increased in stress-exposed WT plants

**Figure 6:**
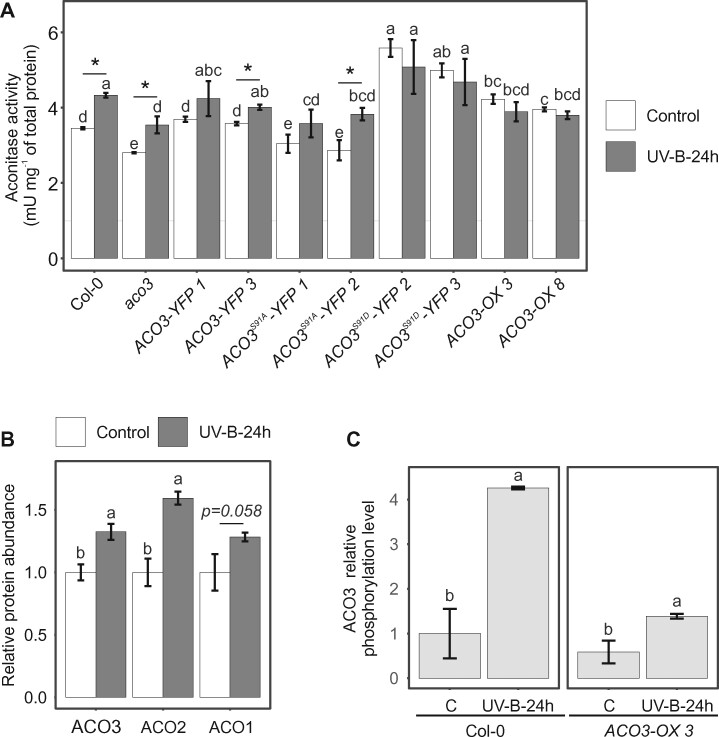
Total foliar ACO activity in WT and *ACO3* mutant plants. A, Total ACO activity in WT (Col-0), *aco3, aco3* expressing *pACO3::ACO3-YFP* (*ACO3-YFP 1* and *ACO3-YFP 3*)*, pACO3::ACO3^S91A^-YFP* (*ACO3^S91A^-YFP 1* and *ACO3^S91A^-YFP 2*), *pACO3::ACO3^S91D^-YFP* (*ACO3^S91D^-YFP 2* and *ACO3^S91D^-YFP 3*), or *35S::ACO3* (*ACO3-OX 3* and *ACO3-OX 8*) in control conditions and 24 h after UV-B treatment (1.5 W m^−2^, 45 min). Values represent the mean of *n* = 3 ± SD. Letters indicate significant differences (Kruskal–Wallis test and Fisher’s least significant difference post hoc test with Bonferroni adjustment, *P* < 0.05). Asterisks indicate statistically significant differences between the control and UV-B-24 h samples for each genotype, Student’s *t* test, *P* < 0.05. B, Relative abundance of ACO 1–3 in *35S::ACO3* (*ACO3-OX 3)* plants 24 h after UV-B treatment (1.5 W m^−2^, 45 min) as measured by PRM. Letters indicate values significantly different from the control sample; Student’s *t* test, *P* < 0.05. Error bars represent SD (*n* = 3). C, tSIM/PRM relative quantification of Ser91 phosphorylation of ACO3 in WT (Col-0) and *35S::ACO3* (*ACO3-OX 3)* plants 24 h after UV-B treatment (1.5 W m^−2^, 45 min). Error bars represent SD (*n* = 3 except in the case of Col-0 control, where *n* = 2). Letters indicate values significantly different from the control for each genotype; Student’s *t* test, *P* < 0.05.

Finally, we tested if alterations in ACO3 abundance and/or phosphorylation corresponded with changes in total foliar ACO activity. Enzymatic assays were first performed with crude leaf extracts isolated from plants grown under control conditions. The phosphomimetic *ACO3^S91D^-YFP 2* and *ACO3^S91D^-YFP 3* lines showed 1.6 and 1.4-fold increases in total ACO activity, respectively, as compared to the WT control plants (Figure 6). Increased total ACO activity was also observed in the *ACO3-OX* lines when compared to WT, but the 1.1- and 1.2-fold increases in the enzymatic activities of *ACO-OX 3* and *ACO-OX 8* were less pronounced when compared to the *ACO3^S91D^-YFP* lines (Figure 6). The *ACO3-YFP* lines showed ACO activities similar to the WT (Figure 6). In contrast, *ACO3^S91A^-YFP 1*, *ACO3^S91A^-YFP 2*, and *aco3* showed 0.9-, 0.8-, and 0.8-fold total ACO activities, respectively, as compared to WT control plants (Figure 6).

When assessed 24 h after a UV-B treatment (1.5 Wm^−2^, 45 min), total ACO activity was increased in WT, *aco3*, *ACO3-YFP*, and *ACO3^S91A^-YFP* lines (Figure 6), which was in line with the stress-induced increase in the abundance of the ACO1-3 isoforms (Figure 4; Supplemental Figure S2). However, *aco3* and the *ACO3^S91A^-YFP* lines did not reach WT levels of stress-induced ACO activity (Figure 6). In contrast to the other genotypes, *ACO3^S91D^-YFP* and *ACO-OX* lines, which already exhibited elevated ACO activity in control conditions, did not show further stress-induced increases in their total ACO activities (Figure 6). This was intriguing, given that both the abundance of ACO1–3 isoforms and the relative level of Ser91 phosphorylation were increased when the *ACO3-OX3* line was exposed to UV-B stress (Figure 6, B and C). The abundance of RBCL, RBCS1A, or GAPA did not show stress-induced changes (Supplemental Figure S3D). Hence, neither the phosphomimetic mutation of *ACO3^S91D^-YFP* nor the 1.4-fold increased relative ACO3 phosphorylation in UV-B exposed *ACO3-OX3* (Figure 6, A and C) promoted increased foliar ACO activity under stress conditions. Taken together, it was evident that the prevalence of stress promoted increased ACO activity in WT plants, but no clear relationship between the enzymatic activity, ACO3 abundance, or its phosphorylation at Ser91 under stress conditions could be established.

### Phosphomimetic mutation at ACO3 Ser91 promotes the abundance and activity of components elicited by the MDR

**Figure 7:**
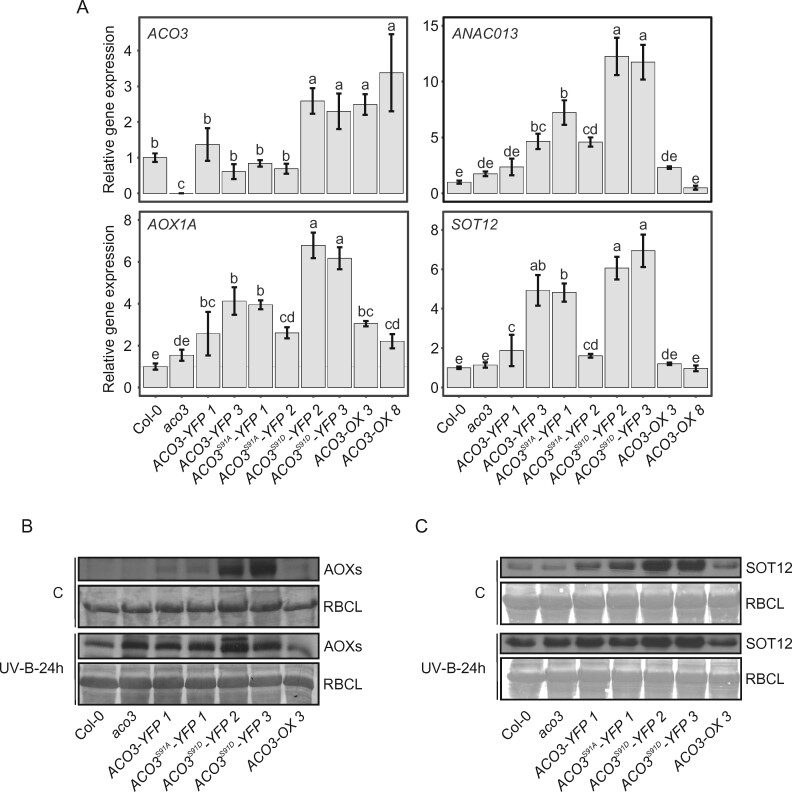
Impact of ACO3-Ser91 phosphomutation on components related to mitochondrial dysfunction. A, RT-qPCR analysis of transcript abundance of *ACO3*, *ANAC013*, *AOX1A*, and *SOT12* in WT (Col-0), *aco3*, *aco3* expressing *pACO3::ACO3-YFP* (*ACO3-YFP 1* and *ACO3-YFP 3*)*, pACO3::ACO3^S91A^-YFP* (*ACO3^S91A^-YFP 1* and *ACO3^S91A^-YFP 2*), *pACO3::ACO3^S91D^-YFP* (*ACO3^S91D^-YFP 2* and *ACO3^S91D^-YFP 3*), or *35S::ACO3* (*ACO3-OX 3* and *ACO3-OX 8*) in normal growth conditions. Error bars represent SE (*n* = 3). Letters indicate significant differences (Kruskal–Wallis test and Fisher’s least significant difference post hoc test with Bonferroni adjustment, *P* < 0.05). B, Protein abundance of AOXs in WT (Col-0), *aco3*, *aco3* expressing *pACO3::ACO3-YFP* (*ACO3-YFP 1*)*, pACO3::ACO3^S91A^-YFP* (*ACO3^S91A^-YFP 1*), *pACO3::ACO3^S91D^-YFP* (*ACO3^S91D^-YFP 2*, and *ACO3^S91D^-YFP 3*) or *35S::ACO3* (*ACO3-OX 3*) in control conditions and 24 h after UV-B treatment (UV-B; 1.5 W m^−2^, 45 min) as detected by immunoblotting. Coomassie staining of RBCL is shown to demonstrate the equal loading of samples. C, Protein abundance of SOT12 in WT (Col-0), *aco3, aco3* expressing *pACO3::ACO3-YFP* (*ACO3-YFP 1*)*, pACO3::ACO3^S91A^-YFP* (*ACO3^S91A^-YFP 1*), *pACO3::ACO3^S91D^-YFP* (*ACO3^S91D^-YFP 2*, and *ACO3^S91D^-YFP 3*) or *35S::ACO3* (*ACO3-OX 3*) in control conditions and 24 h after UV-B treatment (UV-B; 1.5 W m^−2^, 45 min) as detected by immunoblotting. Coomassie staining of RBCL is shown to demonstrate the equal loading of samples.

The ANAC017-dependent increase in ACO3 abundance in stress-exposed plants (Figure 4) raised the question of whether *ACO3* forms a component of the MDR. Therefore, we investigated whether changes in the phosphorylation status and/or abundance of ACO3 were reflected in changes in transcript abundance of *ACO3* or the classical MDR marker genes *ANAC013*, *AOX1A*, or *SOT12* (Figure 7A). The transcript abundance was analyzed by reverse transcription-quantitative PCR (RT-qPCR) in leaves of 4-week-old WT, *aco3*, and transgenic *ACO3-YFP, ACO3^S91A^-YFP*, *ACO3^S91D^-YFP*, and *ACO3-OX* plants grown under normal growth conditions.

Transcript abundance of *ACO3-YFP* and *ACO3^S91A^-YFP* in two independent transgenic lines each was similar to the level of native *ACO3* mRNA in the WT, while *aco3* was devoid of the transcript (Figure 7A). However, *ACO3^S91D^-YFP* lines showed an approximately 2.5-fold increased *ACO3^S91D^-YFP* transcript abundance compared to *ACO3* expression in WT (Figure 7A). The increased ACO3 protein abundance observed in phosphomimetic *ACO3^S91D^-YFP* lines under normal growth conditions, as compared to the other genotypes (Figure 3B), was therefore likely due to transcriptional activation of the native *ACO3* promoter (Figure 1). *ACO3-OX 3* and *AXO3-OX 8* lines, expressing *ACO3* under cauliflower mosaic virus (35S) gene promoter, showed 2.5- and 3.5-fold *ACO3* transcript levels compared to WT (Figure 7A).

Transcript abundance of the ANAC017 target genes *ANAC013*, *AOX1A*, and *SOT12* was significantly higher in lines expressing the phosphomimetic ACO3^S91D^-YFP when compared to all the other genotypes (Figure 7A). The ACO3-OX lines showed increased transcript abundance for *AOX1A*, but not for *ANAC013* or *SOT12* (Figure 7A). Moreover, the two independent transgenic lines expressing *ACO3-YFP* or *ACO3^S91A^-YFP* showed varying increases in the abundance of these stress-related transcripts (Figure 7A). In contrast, transcriptional activation of the MDR marker genes was not observed in the *aco3* knock-out mutant (Figure 7A). These results suggested that phosphorylation of ACO3 was connected with increased transcriptional activation of the MDR.

To investigate the MDR on protein level, we analyzed AOX or SOT12 abundance by immunoblot analysis of plants grown under control conditions. All the YFP-fusion lines, expressing either *ACO3-YFP*, *ACO3^S91A^-YFP*, or *ACO3^S91D^-YFP*, displayed slightly increased abundance of AOXs and SOT12 as compared to the other genotypes (Figure 7, B and C; for data on second independent transgenic lines, see Supplemental Figure S2, B and C). Notably, however, the lines expressing the phosphomimetic ACO3^S91D^-YFP showed higher AOX and SOT12 abundance as compared to those expressing ACO3-YFP or ACO3^S91A^-YFP (Figure 7, B and C;Supplemental Figure S2). No substantial differences in AOX or SOT12 abundance were detected between WT, *aco3*, and the *ACO3-OX* lines (Figure 7, B and C; Supplemental Figure S2, B and C). Following UV-B-induced stress, all genotypes displayed increased levels of AOXs and SOT12 (Figure 7, B and C).

AOXs affect photosynthesis by providing an alternative electron sink for the chloroplast photosynthetic electron transfer chain. To assess the physiological relevance of AOX accumulation in response to the ACO3 phosphorylation status (Figure 7B;Supplemental Figure S2B), we addressed the effect of AOX on photosynthetic electron transfer by imaging kinetics of chlorophyll fluorescence (Fs) under light in leaf discs, as described in Shapiguzov et al. (2019). No difference in Fs was observed between the genotypes in mock-treated controls. However, when AOX activity was inhibited by salicylhydroxamic acid (SHAM), a reproducibly elevated Fs was recorded in the ACO3^S91D^-YFP lines, as compared to all other tested genotypes (Supplemental Figure S6). This suggested that AOX function could prevent over-reduction of the photosynthetic electron transfer chain in the ACO3^S91D^-YFP lines. When SHAM was applied together with methyl viologen (MV), a rise in Fs that peaked about 40 min after the start of the light treatment was observed. This Fs rise is associated with accumulation of reducing equivalents in the photosynthetic electron transfer chain in the situation where AOX activity is inhibited (Shapiguzov et al., 2019). Such elevated Fs could rise because of parallel regulatory adjustments occurring on the thylakoid membrane. Recent studies on plants with constitutively active MDR provided evidence that increased AOX activity coincided with increased activity of the chloroplast NAD(P)H dehydrogenase (NDH) complex, which is involved in cyclic electron transfer around Photosystem I (Shapiguzov et al., 2020). Hence, when AOX activity is pharmacologically inhibited by SHAM, enhanced chloroplast NDH activity could promote reduction of the plastoquinone pool and lead to the observed elevation in Fs. All *ACO3* complementation lines demonstrated a higher Fs increase when compared to the WT or *aco3*. However, the highest Fs increase developed in the two independent *ACO3^S91D^-YFP* lines (Supplemental Figure S6), providing further evidence for elevated AOX function in the *ACO3^S91D^-YFP*-expressing plants.

### ACO3 contributes to tolerance against UV-B or AA-induced stress

**Figure 8:**
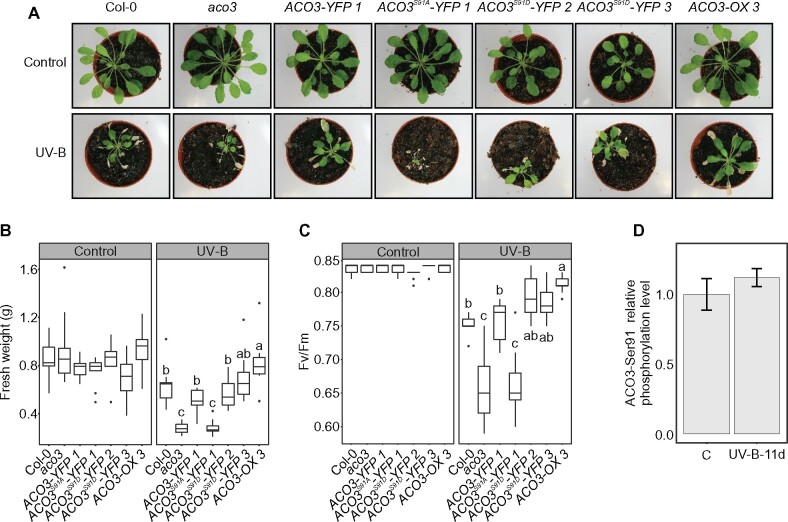
Importance of ACO3 and its regulation by reversible phosphorylation at Ser91 in tolerance to UV-B-induced mitochondrial dysfunction. A, Phenotypes of WT (Col-0), *aco3*, *aco3* expressing *pACO3::ACO3-YFP* (*ACO3-YFP 1*)*, pACO3::ACO3^S91A^-YFP* (*ACO3^S91A^-YFP 1*), *pACO3::ACO3^S91D^-YFP* (*ACO3^S91D^-YFP 2* and *ACO3^S91D^-YFP 3*), or *35S::ACO3* (*ACO3-OX 3*), 11 d after UV-B treatment (1.5 W m^−2^, 45 min). The untreated control plants were kept under growth light. B, Fresh weights of the control and UV-B exposed plants 11 d after the treatment (*n* = 9). Letters indicate significant differences (one-way ANOVA with Tukey’s HSD test, *P* < 0.05). C, Maximum efficiency of PSII (Fv/Fm) of the control and UV-B exposed plants 11 d after the treatment (*n* = 9). Letters indicate significant differences (Kruskal–Wallis test and Fisher’s least significant difference post hoc test with Bonferroni adjustment, *P* < 0.05). D, tSIM/PRM relative quantification of ACO3 Ser91 phosphorylation in WT (Col-0) plants in control conditions and 11 d after UV-B treatment (1.5 W m^−2^, 45 min). Error bars represent SD (*n* = 3). B and C, The box plots show the median (central line), the lower and upper quartiles (box), the minimum and maximum values within 1.5× the interquartile range (whiskers), and the outliers (points).

To explore the potential role of ACO3 and its posttranslational regulation by phosphorylation at Ser91 in plant stress tolerance, WT plants, and the different *ACO3* mutants were subjected to UV-B stress. Four-week-old plants were exposed to a high-irradiance UV-B dose (1.5 Wm^−2^, 45 min) and their recovery from the abrupt stress treatment was monitored (Figure 8A; for data on independent transgenic lines, see Supplemental Figure S2D). Eleven days after the 45 min UV-B treatment, *aco3* and *ACO^3S91A^-YFP* displayed decreased photosynthetic performance (Fv/Fm) and reduced rosette fresh weight, while the opposite was recorded for *ACO3-OX* plants (Figure 8, B and C;Supplemental Figure S2, E and F). Hence, we found that *aco3* and *ACO^3S91A^-YFP* lines were more susceptible and *ACO3-OX* was more tolerant to UV-B stress than the WT (Figure 8;Supplemental Figure S2D–F). The *ACO3^S91D^-YFP* lines, which mimic constitutive phosphorylation at Ser91, did not show increased tolerance to UV-B-induced stress (Figure 8;Supplemental Figure S2D–F), suggesting that the ability to reversibly control Ser91 phosphorylation appears to be required to obtain the benefits from overexpressing ACO3. Consistently, while the level of ACO3-Ser91 phosphorylation increased in response to stress (Figure 4, C and D**)**, it did not differ from WT control levels when measured 11 d after the stress treatment (Figure 8D).

When germinated on one-half Murashige and Skoog (MS) supplemented with AA, growth of *aco3* and the nonphosphorylatable *ACO^3S91A^-YFP* mutants was diminished as compared to WT or the *ACO3-YFP* complementation line (Supplemental Figure S7). The *ACO3^S91D^-YFP* lines did not significantly differ from WT, whereas *ACO3-OX* grew better than the other genotypes when exposed to the pharmacological stress agent (Supplemental Figure S7). Thus, both the abundance and the phosphorylation status of ACO3 are connected with tolerance to mitochondrial dysfunction.

### ACO3 Ser91 is present in gymnosperms and Brassicaceae

**Figure 9:**
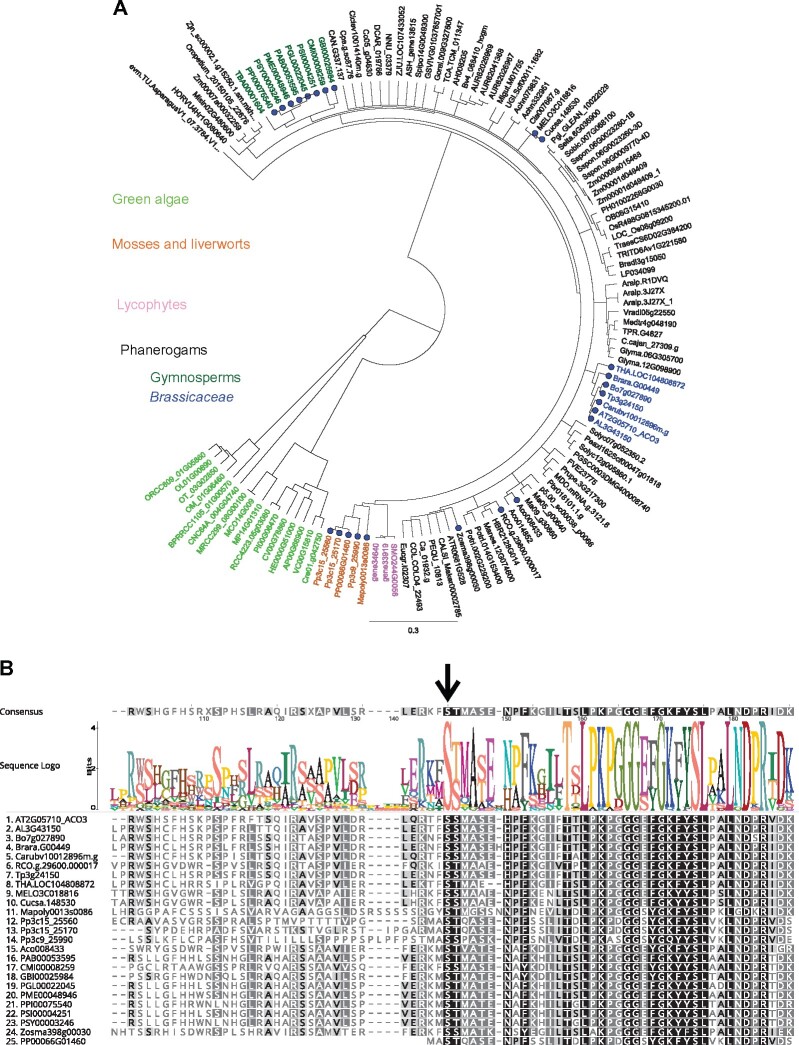
Analysis of ACO3 Ser91 conservation in plants. A, Phylogenetic tree of ACO3 orthologs in major plant lineages. Blue dots indicate those species in which a Ser corresponding to Arabidopsis Ser91 was found. B, Conservation of the amino acid sequences surrounding Ser91 (black arrow) in those species in which it was found to be conserved. Color scale indicates amino acid similarity according to the BLOSUM62 similarity scoring matrix using a threshold of 1 (100% similar, black; 80%–100% similar, dark grey; 60%–80% similar, light grey; ˂60% similar, uncolored). For A and B, The abbreviations indicate different species: *Actinidia chinensis* (Achn), *Amaranthus hypochondriacus* (AH), *Amborella trichopoda* (ATR), *A. comosus* (Aco), *Apostasia shenzhenica* (ASH), *Arabydopsis lyrata* (AL), *Arabidopsis thaliana* (AT), *Arachis ipaensis* (Araip), *Asparagus officinalis* (evm.TU.AsparagusV1), *Bathycoccus prasinos* (BPRRCC1105), *Brachypodium distachyon* (Bradi), *Beta vulgaris* (Bv), *Brassica oleracea* (Bo), *Brassica rapa* (Brara), *Cajanus cajan* (C.cajan), *Calamus simplicifolius* (CALSI), *Capsella rubella* (Carbv), *Capsicum annuum* (CAN), *Carica papaya* (Cpa), *Cenchrus americanus* (Pgl), *Chenopodium quinoa* (AUR), *Chlamydomonas reinhardtii* (Cre), *Chlorella sp.* NC64A (CNC64A), *Citrullus lanatus* (Cla), *Citrus clementina* (Ciclev), *Coccomyxa sp.* C169 (CV), *Coffea canephora* (Cc), *Corchorus olitorius* (COL), *C. melo* (MELO), *C. sativus* (Cucsa), *Cycas micholitzii* (CMI), *Daucus carota* (DCAR), *Elaeis guineensis* (p5.00), *Erythranthe guttata* (Migut), *Eucalyptus grandis* (Eucgr), *Fragaria vesca* (FVE), *Ginkgo biloba* (GBI), *Glycine max* (Glyma), *Gossypium raimondii* (Gorai), *Hevea brasiliensis* (HBR), *Hordeum vulgare* (HORVU), *Lolium perenne* (LP), *Malus domestica* (MDO), *Manihot esculenta* (Manes), *M. polymorpha* (Mapoly), *Medicago truncatula* (Medtr), *Micromonas commoda* (MCO), *Micromonas pusilla* strain CCMP1545 (MP), *Micromonas sp.* RCC299 (MRCC299), *Musa acuminata* (Ma), *Nelumbo nucifera* (NNU), *Ostreococcus lucimarinus* (OL), *Ostreococcus sp.* RCC809 (ORCC809), *Ostreococcus tauri* (OT), *Oropetium thomaeum* (Oropetium), *Oryza sativa ssp*. *indica* (Os), *Oryza sativa ssp*. *japonica* (LOC_Os), *Petunia axillaris* (Peaxi), *Phalaenopsis equestris* (PEQU), *Phyllostachis edulis* (PHO), *P. patens* (Pp, PP), *Picea abies* (PAB), *Picea glauca* (PGL*), Picea sitchensis* (PSI), *Pinus pinaster* (PPI), *Pinus sylvestris* (PSY), *Pinus taeda* (PTA*), Populus trichocarpa* (Potri), *Prunus persica* (Prupe), *Pseudotsuga menziesii* (PME), *Pyrus bretschneideri* (Pbr), *R. communis* (RCO), *Saccharum spontaneum* (Sspon)*, Schrenkiella parvula* (Tp), *Selaginella moellendorffii* (gene, SMO), *Setaria italica* (Seita), *Solanum lycopersicum* (Solyc), *Solanum tuberosum* (PGSC), *Sorghum bicolor* (Sobic), *Tarenaya hassleriana* (THA), *Taxus baccata* (TBA), *Thalassiosira pseudonana* (Tp), *Theobroma cacao* (TCA), *Trifolium pratense* (TPR), *Triticum aestivum* (Traes), *Triticum turgidum* (TRITD), *Utricularia gibba* (UGI), *Vigna radiata var*. *radiata* (Vradi), *Vitis vinifera* (GSVIVG), *Volvox carteri* (VC), *Zea mays* (Zm), *Ziziphus jujuba* (ZJU.LOC), and *Z. marina* (Zosma).

Since little is known about posttranslational control of metabolic adjustments in mitochondrial retrograde signaling and stress tolerance, we investigated the conservation of the ACO3 Ser91 residue in species from the major branches of the plant lineages. A phylogenetic tree was constructed based on a transitive consistency score (TCS) weight-curated M-Coffee multiple sequence alignment of Arabidopsis ACO3 orthologs from different plant species, as identified by combined comparative genomics in Dicots PLAZA version 4.0, Monocots PLAZA version 4.5, Gymno PLAZA version 1.0 and “Green Plants” in pico-PLAZA version 3.0 databases (Figure 9A;Supplemental Data Set 1).

The presence of a Ser residue at a site corresponding to the Arabidopsis ACO3 Ser91 turned out to be particularly characteristic of gymnosperms as well as species within the family of *Brassicaceae* (Figure 9A), although the Ser residue was also found in other angiosperm species, including melon (*Cucumis melo*), cucumber (*Cucumis sativus*), eelgrass (*Zostera marina*), castor bean (*Ricinus communis*), and pineapple (*Ananas comosus*; Figure 9A). The Ser residue was also present in the liverwort *Marchantia polymorpha* and the moss *Physcomitrium* (*Physcomitrella*) *patens*, which represent lineages among early land plants. Furthermore, the amino acid sequences surrounding the site corresponding to Ser91 in ACO3 exhibited a significant degree of conservation and comprised an R-x-x-S motif, which is a widely conserved eukaryotic phosphorylation motif (Figure 9B; Bradley and Beltrao, 2019). Such sequence conservation is necessary for accurate recognition of the phosphorylation site by regulatory protein kinases or phosphatases, and supports the conclusion that Ser91 is a phosphosite with a regulatory function (Miller and Turk, 2016; Trost et al., 2016).

## Discussion

Mitochondrial energy metabolism is an integral component of the metabolic and regulatory networks that maintain basic cellular functions and allow survival under environmental challenges. The best-known mitochondrial retrograde signaling pathway involves stress-induced activation and translocation of the transcription factors ANAC013 and ANAC017 from the ER to the nucleus, where they induce gene expression changes in response to mitochondrial dysfunction (De Clercq et al., 2013; Ng et al., 2013; Meng et al., 2019). Major gaps remain in understanding how the transcriptomic adjustments translate into appropriate metabolic changes that protect the plant against excessive cellular damage. Here we propose a role for ACO3 as a component of the MDR in Arabidopsis.

### ACO3 contributes to plant stress tolerance

Proteomic studies have identified mitochondrial enzymes that undergo posttranslational modifications (PTMs), which provide fast and efficient means for metabolic regulation upon environmental changes (reviewed by Møller et al., 2020). In most cases, however, the molecular machineries underlying the variety of PTMs and the physiological significance of their regulatory actions remain unknown. A previous study proposed a regulatory interaction between ACO3 and a PP2A regulatory subunit PP2A-B’γ, which interacted with ACO3 in the cytosol and affected its phosphorylation at Ser91, a phosphorylation site unique to the ACO3 isoform (Konert et al., 2015). This protein interaction was discussed in terms of negative regulation of stress responses, but the role of ACO3 phosphorylation remained elusive. Here we set out to specify the impact of ACO3 phosphorylation at Ser91 in plant stress tolerance. Confocal imaging of transgenic Arabidopsis lines expressing *ACO3-YFP*, *ACO3^S91A^-YFP*, or *ACO3^S91D^-YFP* implied that the substitutions of Ser91 did not disrupt mitochondrial import of the protein, which gave strong fluorescence signals in leaf mitochondria in both control and UV-B stress conditions (Figure 2).

ACOs are tightly embedded in the plant cell’s metabolic networks and their activities are critical in several metabolic circuits. By catalyzing an enzymatic step as part of the TCA cycle, ACOs contribute to the availability of ATP, isocitrate, and other down-stream metabolites in mitochondria (Carrari et al., 2003; Taylor et al., 2004). In germinating seeds, ACOs enable the production of carbohydrates from fatty acids in the glyoxylate cycle (Hooks et al., 2014). ACOs also supply substrate for cytosolic isocitrate dehydrogenases and thereby contribute to the formation of NADPH and 2-oxoglutarate, the latter of which serves as a substrate for glutamate and glutamine biosynthesis in the cytosol (Mhamdi et al., 2010). Therefore, cytosolic ACOs are indispensable for ammonium fixation, especially under conditions that promote photorespiration and, through cytosolic glutamine synthase activity, the associated nitrate assimilation.

While the metabolic role of ACO3 in stress-exposed tissues remains to be established, it seems clear that the enzyme is an important contributor to plant stress tolerance and must therefore be flexibly coordinated within regulatory networks. Our results imply that the lowered ACO activity, as observed in the *aco3* knockout and the *ACO3^S91A^-YFP* lines, associated with susceptibility to AA or UV-B-induced stress, while the opposite took place in plants over-expressing ACO3 (Figures 6, 8;Supplemental Figures S2, S7). Intriguingly, the *ACO3^S91D^-YFP* lines, in which the mutation mimicked constitutive phosphorylation of ACO3 and did not allow dephosphorylation of the protein, did not show increased stress tolerance when compared to WT (Figure 8**;**Supplemental Figures S2 and S7). This finding suggested that *ACO3* over-expression, together with the ability to reversibly phosphorylate Ser91, were prerequisites for increased stress tolerance in Arabidopsis leaves. On the other hand, the obvious discrepancies between ACO abundance, level of ACO3 phosphorylation, and the extractable ACO activities (Figures 4, 6) imply that foliar ACO activity may be governed by yet another, so-far unknown, posttranslational mechanism of regulation.

### ACO3 and its posttranslational regulation respond to mitochondrial dysfunction

Appropriate regulation of mitochondrial functions is vital for plant performance, as evidenced by the severe growth phenotypes observed in mutants suffering from constitutive mitochondrial dysfunction or impaired mitochondrial retrograde signaling (Van Aken et al., 2016). The exact chemical nature of mitochondrial stress signals remains enigmatic, but metabolic intermediates, calcium, and ROS, arising as a consequence of mitochondrial perturbations, have been discussed (Van Aken et al., 2016).

Over recent years, meta-analysis of publicly available transcript profiles has provided insights into common and specific transcriptional responses to various stress conditions and identified target genes that reflect the activation of MDR (Van Aken and Whelan, 2012; De Clercq et al., 2013; Ng et al., 2013). De Clercq et al. (2013) uncovered a set of 24 genes, including *AOX1A* and *SOT12*, which were strongly transcriptionally induced in several mitochondrial perturbation experiments, suggesting they represent robust marker genes for MDR. The gene set reflected stress-induced activation of various protective functions, including detoxification, protection of photosynthetic apparatus, chaperone-like functions, transport of xenobiotics, and proteasomal degradation of proteins (De Clercq et al., 2013; Meng et al., 2019; Shapiguzov et al., 2019).

Analysis of gene promoters revealed that the 24 identified MDR genes shared a cis-regulatory element termed mitochondrial dysfunction motif (MDM), which shares elements with other stress-responsive pathways. For example, the MDM overlaps with two promoter elements that are required for UV-B-induced transcriptional reprogramming (Safrany et al., 2008; De Clercq et al., 2013). Therefore, different regulatory pathways may converge at the promoter elements to integrate signals from various subcellular sources. The MDM-containing genes are under the control of ANAC017 and their transcript abundance becomes strongly increased during severe mitochondrial dysfunction (De Clercq et al., 2013, Ng et al., 2013). In the nucleus, *in vivo* interactions between ANAC013/ANAC017 and the negative regulator RCD1 modulate the expression of a subset of MDR genes, including those encoding AOX1A and SOT12 (Shapiguzov et al., 2019). *AOX1A* and *SOT12* show very low expression levels in the absence of stress signals, and their transcript and protein abundance becomes strongly increased in response to environmental, pharmacological, or genetic perturbations (De Clercq et al., 2013; Shapiguzov et al., 2019; Figure **7**). Although the role of MDR in eliciting cellular detoxification appears clear, little is known about metabolic regulation under mitochondrial dysfunction.

We found that both ACO3 abundance and the relative level of its phosphorylation at Ser91 increased in response to AA or UV-B-induced stress and that these regulatory adjustments required signaling through ANAC017 (Figure 4). Pharmacological approaches with the protein synthesis inhibitor CHX further implied that the UV-B-induced increases in ACO3 abundance and phosphorylation were only partially dependent on *de novo* protein synthesis (Figure 5). The protein kinase responsible for ACO3 phosphorylation and the subcellular localization of the regulatory action, however, remains unresolved. Given that the ACO3 phosphorylation status was clearly reflected in the abundance of MDR-related transcripts (Figure 7), it is possible that ACO3 phosphorylation at Ser91 affects its moonlighting activities in the regulation of gene expression, rather than its enzymatic activity within metabolic networks.

Reciprocally to the stress-induced increase in ACO3-Ser91 phosphorylation, manipulation of the ACO3 phosphorylation status at Ser91 triggered increased transcript abundance of *ACO3* itself, as well as *ANAC013*, *SOT12*, and *AOX1A*, which was reflected in accumulation of ACO3, AOX, and SOT12 on protein level (Figures 3 and 7). The increased *ACO3* transcript abundance could relate to ANAC017 function, as the *ACO3* gene promoter carries MDMs putatively recognized by ANAC017 (De Clercq et al., 2013; Supplemental Data Set 2). Moreover, *ACO3* transcript abundance was elevated in *ANAC017* over-expressing plants (Meng et al., 2019). Thus, posttranslational regulation of ACO3 by phosphorylation of Ser91 appeared both as a target and mediator of the mitochondrial dysfunction signaling.

Evidence for ACO-dependent mitochondrial signaling has also been obtained by pharmacological approaches. Inhibition of ACO activity in Arabidopsis leaves led to activation of MDR in a ROS-independent manner, suggesting the involvement of metabolic retrograde signaling (Umbach et al., 2012), which is in line with the minute ROS accumulation observed in the transgenic *ACO3* lines (Figure 1C). In Arabidopsis and tobacco, accumulation of citrate was found to be an important signal to induce AOX, by either direct addition of citrate to suspension cultures or by inhibition of ACO activity by monofluoroacetate (Vanlerberghe and Mclntosh, 1996; Gray et al., 2004; Clifton et al., 2005; Finkemeier et al., 2013).

Our findings suggest that posttranslational regulation of ACO3 in Ser91 is connected to the activation of the MDR in Arabidopsis. The residue corresponding to Arabidopsis ACO3-Ser91 is not widely conserved among major plant lineages, but appears particularly characteristic of gymnosperms and *Brassicaceae* (Figure 9A). Despite its presence in the moss *P. patens* and the liverwort *M. polymorpha*, the residue was not consistently observed in lycophytes or phanerogams, suggesting that posttranslational regulation of ACO3 by phosphorylation at Ser91 may have evolved independently several times during the evolution of land plants.

## Materials and methods

### Plant material and growth conditions

*Arabidopsis thaliana* Columbia (Col) WT, *aco3* T-DNA mutant line (SALK_013368, previously characterized in Arnaud et al., 2007), *anac017-1* (SALK_022174; Ng et al., 2013), and *rcd1* (GK-229D11; Shapiguzov et al., 2019) were used in the experiments. Plants were grown in 8/16 h light/dark period under 120 μmol photons m^−2^ s^−1^ at 23°C and 50% relative humidity.

### Construction of transgenic lines

The native promoter region of *ACO3* was determined using AtcisDB (http://agris-knowledgebase.org/AtcisDB/), and a 2631 nucleotide region upstream of the translational start codon was cloned in front of mutated and WT *ACO3* coding sequences. The *ACO3* promoter and the coding sequence were cloned without the stop codon into pGREENII0029 vector containing a YFP sequence. The primers and restriction enzymes used in the molecular cloning are listed in Supplemental Table S1. Mutagenesis of Ser91 of ACO3 was carried out with QuickChange II XL Site-Directed Mutagenesis Kit (Agilent Technologies, Santa Clara, CA, USA) using the primers listed in Supplemental Table S1. For generation of *ACO3* overexpression lines (*ACO3-OX*), the *ACO3* coding sequence was amplified and cloned into pGREENII0029 vector under the cauliflower mosaic virus (35S) gene promoter. Additional start codon present in the multiple cloning sites of the pGREENII0029 vector in *ACO3-OX* construct was removed by mutagenesis with QuickChange II XL Site-Directed Mutagenesis Kit (Agilent Technologies) using primers listed in Supplemental Table S1. The gene constructs were verified by sequencing, and transformed into Arabidopsis *aco3* mutant background by floral dipping. Multiple T1 plants were selected by their kanamycin resistance on antibiotic selection plates and analyses were performed with homozygous T3 plants.

### Reverse transcription quantitative PCR

For analysis transcript abundance, RNA was isolated from 4-week-old Arabidopsis rosettes with innuPREP Plant RNA Kit (no. 845-KS-2060250; Analytik Jena AG, Jena, Germany) and thereafter DNase-treated with an Ambion Turbo DNA-free Kit (no. AM1907; Thermo Fisher Scientific Waltham, MA, USA) according to the manufacturer’s instructions for rigorous DNase treatment. One microgram of RNA was used for the synthesis of cDNA with the RevertAid First Strand cDNA Synthesis kit (no. K1622; Thermo Fisher Scientific, Waltham, MA, USA). RT-qPCR was performed in an iQ5 Real-Time PCR system (Bio-Rad, Hercules, CA, USA) using PowerUp SYBR Green master mix (no. A25777; Thermo Fisher Scientific, Waltham, MA, USA). Normalized Relative Quantities (NRQs) and Standard Errors of NRQ were determined according to Hellemans et al. (2007). The reference genes described in Czechowski et al. (2005) were assayed in the sample set. The polyubiquitin genes *At5g25760 (UBC)* and *At4g05320 (UBQ10)* were determined to have the most stable expression and were used for normalization, as in Vandesompele et al. (2002). The primers are listed in Supplemental Table S1.

### Analysis of stress tolerance

For germination tests, seeds were sterilized in 70% (v/v) ethanol, 0.5% Triton X-100 (v/v) for 3 min and thereafter washed twice with 95% (v/v) ethanol. Seeds were sown on 50% MS solid medium (Sigma-Aldrich St Louis, MO, USA; M5524; Murashige and Skoog, 1962) on Petri dishes. To study the germination capacity under mitochondrial dysfunction stress, the MS medium was supplemented with 50 μM of AA. After 3 d at 4°C in darkness, the plates were transferred to 120 μmol photons m^−2^ s^−1^ at 8/16 h light/dark period under 23°C. The fresh weight of the seedlings was measured after 1 week of growth.

Stress treatments were performed on 4-week-old plants grown under control conditions of 120 μmol photons m^−2^ s^−1^ at 23°C and 50% relative humidity. UV-B stress was applied with Philips Broadband ultraviolet-B lamp (TL20W/12 RS; Philips, Amsterdam, Netherlands) at an irradiance of 1.5 W m m^−2^ for 45 min. Thereafter, the plants were transferred to the control growth conditions, and samples were collected 24 h into the recovery period. AA was applied by spraying the rosettes with 50 μM AA in 0.01% (v/v) Tween-20, and samples were taken after a 10-h treatment under control growth conditions. For mock treatment, the plants were sprayed with 0.01% (v/v) Tween-20. The rosettes were harvested in pools of three, immediately frozen in liquid N_2_ and stored at −80°C until protein isolation.

### Measurement of chlorophyll fluorescence

Maximum efficiency of PSII (Fv/Fm) was measured in excised leaf discs using an Open FluorCam FC 800-O/1010 following the manufacturer’s instructions (Photon System Instruments, Drásov, Czech Republic). For measurements of photosynthesis in relation to AOX functions, leaf discs were soaked in water supplemented with 0.05% (v/v) Tween-20 with or without 1 µM MV and/or 2 mM SHAM. An equal volume of DMSO was used in SHAM-free controls. Leaf discs were dark-adapted for 1 h to allow for the uptake of the chemicals. The kinetics of chlorophyll Fs in low-intensity light (80 µmol photons m^−2^ s^−1^) were recorded using an Imaging PAM (Walz, Effeltrich, Germany).

### Histochemical analysis of H_2_O_2_ accumulation

Accumulation of H_2_O_2_ was studied using DAB (Sigma-Aldrich) staining as previously described in Kangasjarvi et al. (2008).

### Microscopy

Detached leaves from the transgenic YFP-fusion lines were imaged with the following microscope settings: Microscope Zeiss LSM780; objective C-Apochromat 40×/1.20 W Korr M27; beam splitters MBS 458/514 for YFP, MBS 458/543 for Mitotracker, MBS 488/543/633 for Chlorophyll A; zoom 2.0. YFP fluorescence was excited with 514 nm light and the emission was monitored at 519–550 nm. Chlorophyll A fluorescence was excited with 633 nm light and the fluorescence was monitored at 647–721 nm. Mitotracker Orange CMTMRos (Invitrogen, Carlsbad, CA, USA) was applied as 1 μM solution infiltrated into leaves before imaging, and excited with 543 nm light and detected at 551–604 nm. Laser intensities for YFP, Mitotracker and Chlorophyll A were 3.0%, 2.0%, and 2.2% for Figure 2A; 4.5%, 2.6%, and 1.5% for Figure 2B; and 5.5%, 3.5%, and 1.5% for Supplemental Figure S1, respectively. Pinhole was 111 µm for Figure 2A; 78 µm for Figure 2B; and 157 µm for Supplemental Figure S1. Gain for YFP, Mitotracker, and Chlorophyll A was 781, 759, and 621 for Figure 2A; 832, 817, and 689 for Figure 2B; and 815, 730, and 689 for Supplemental Figure S1, respectively. Images were acquired using ZEN 2.3 SP1 black edition (Carl Zeiss, Oberkochen, Germany). Single images were created with Zen 2.1 (black) software version 11,0,0,190 (Carl Zeiss). The minimum and maximum intensities of fluorescence signal were adjusted using Zen 2.1 (black) software version 11,0,0,190 (Carl Zeiss) to correct for differences in signal intensity between different fluorophore channels (YFP, Mitotracker, and Chlorophyll A). For Supplemental Figure S1, micrographs were cropped using Zen 2.1 (black) software version 11,0,0,190 (Carl Zeiss) to better visualize cellular structures and subcellular localization of YFP.

### Protein synthesis inhibitor assay

Four-week-old plants were treated by spraying with 25 µM CHX in 0.01% (v/v) Tween-20 or 0.01% (v/v) Tween-20 (mock). A subset of plants from each treatment was treated 2 h later with UV-B (1.5 W m m^−2^ for 45 min). Thereafter, the plants were transferred to the control growth conditions and samples were collected 24 h into the recovery period. The rosettes were harvested in pools of three, immediately frozen in liquid N_2_ and stored at −80°C until protein isolation.

### Protein quantification by targeted mass spectrometry

Protein from 4-week-old Arabidopsis rosettes was isolated by grinding the leaf material in 50 mM HEPES KOH pH 7.8, 10 mM MgCl_2_ supplemented with protease (Complete-Mini, Roche, Basel, Switzerland) and phosphatase (PhosSTOP; Roche) inhibitor cocktails. Protein was quantified with Protein Assay Dye Reagent (no. 5000006, Bio-Rad, Hercules, CA, USA). The individual ACO1 (AT4G35830), ACO2 (AT4G26970), and ACO3 (AT2G05710; www.arabidopsis.org) isoforms were quantified by PRM. Samples consisting of 25 µg of protein were prepared for MS analysis as described in Trotta et al. (2019). The trypsin-digested samples were first spiked with iRT peptides (Biognosys, Zürich, Switzerland) according to the manufacturer’s instructions and analyzed in Data Dependent Acquisition (DDA) mode, selecting the top 20 most intense precursors in each scan (*m*/*z* 300–2,000) for higher-energy collisional dissociation fragmentation with an exclusion window of 12 s. The analysis was performed in a nanoflow HPLC system (EasyNanoLC1000; Thermo Fisher Scientific) equipped with a 20 × 0.1 mm^2^ i.d. precolumn combined with a 150 mm × 75 µm i.d. analytical column, both packed with 5 µm Reprosil C18-bonded silica (Dr Maisch GmbH, Ammerbuch, Germany), and injection to an electrospray ionization source coupled to a Q‐Exactive (Thermo Fisher Scientific) mass spectrometer. The mobile phase consisted of water/acetonitrile (ACN; 98:2 (v/v)) with 0.1% formic acid (FA; v/v; solvent A) or ACN/water (80:20 (v/v)) with 0.1% FA (v/v; solvent B) at a flow rate of 300 nL min^−1^. The peptides were separated by a three-step elution gradient: from 3% (v/v) to 43% (v/v) solvent B in 45 min, followed by an increase to 100% (v/v) in 5 min, and 10 min of 100% (v/v) solvent B. In parallel, foliar extracts consisting of 100 µg of protein were separated on a 10% (w/v) SDS-PAGE gel and a protein band migrating at the Arabidopsis ACO molecular weight (100 kDa) was excised and processed and analyzed by MS in the same way.

The resulting tandem MS spectra were searched in Proteome Discoverer 2.3 (Thermo Scientific) with a nonredundant Arabidopsis proteome (TAIR10, www.arabidopsis.org) appended with the most common contaminants using Mascot (version 2.7; Matrix Science; Supplemental Tables S2 and S3). Monoisotopic mass, a maximum of two missed cleavages, 10 ppm precursor mass tolerance, 0.02 Da fragment mass tolerance, and charge ≥2+ were used as settings for the searches. Additionally, methionine oxidation and serine, threonine, and tyrosine phosphorylation were considered as variable modifications, and cysteine carbamidomethylation as fixed modifications. Identification confidence was validated through PhosphoRS filter and Decoy Database Search using 0.01% (strict) and 0.05% (relaxed) false discovery rate confidence thresholds. These data were used for construction of a reference library for the performance of label-free relative quantification of ACO1–3 by PRM. Three or four proteotypic peptides per protein were selected from the reference library, avoiding peptides with potential PTMs (Supplemental Table S4). PRM analysis was performed in a Q-Exactive HF mass spectrometer (Thermo Fisher Scientific) using the same elution gradient described above. PRM data were analyzed with Skyline (Maclean et al., 2010). Comparability between samples was ensured by injecting an equal amount of digested protein (100 µg). In addition, in stress experiments, the abundances of RBCL (ATCG00490), RBCS1A (AT1G67090), and GAPA (AT3G26650) were monitored in parallel with the ACO isoforms. Relative protein abundance was calculated as the sum of the intensity of the three proteotypic peptides targeted for each protein, and calculated as the sum of the integrated peak area of the three most intense fragments with the following formula:

(1) $$A=\left(f11+f12+f13\right)+\left(f21+f22+f23\right)+(f31+f32+f33)$$

where *A* is the relative protein abundance, *f*11, *f*12… are the integrated areas of the most intense ion fragments of peptide 1; *f*21, *f*22… the integrated areas of the most intense ion fragments of peptide 2, and so on. Relative protein abundance values were expressed relative to WT.

The quantification of ACO3 Ser91 phosphorylation was performed by tSIM/PRM. The spectral libraries generated for the PRM analysis of ACO3 in this study and the libraries reported by Konert et al. (2015) were used as references. Foliar extracts consisting of 150 µg of protein were first separated on a 10% SDS-PAGE gel. The protein band migrating at 100 kDa and containing the Arabidopsis ACO isoforms was excised and prepared for MS analysis as described in Trotta et al. (2019). The analysis was performed in a nano HPLC system (EasyNanoLC1000; Thermo Fisher Scientific) coupled to a Q-Exactive HF (Thermo Fisher Scientific). The peptides were separated using the same elution gradient described above. The stoichiometry of ACO3-Ser91 phosphopeptide was calculated in Trotta et al. (2019) and Secher et al. (2016) as the percentage of the intensity of the phosphopeptide over the sum of the intensities of the nonphosphorylated peptide plus the phosphopeptide.

The following formula was used:

(2) $$P=\frac{\left(pt1+pt2+pt3+pt4\right)}{\left(\left(t1+t2+t3\right)+\left(pt1+pt2+pt3+pt4\right)\right)}\;\times \;100\;$$

where *P* is the relative level of phosphorylation, *pt*1, *pt*2… are the integrated areas of the most intense peptide fragments of the phosphorylated form, and *t*1, *t*2… are the integrated areas of the most intense fragments of the nonphosphorylated form. Both the doubly and triply charged nonphosphorylated peptides were targeted. The intensities of both forms were summed to get the peptide total intensity. The percentage of phosphorylation in all the analyzed genotypes and experimental conditions was obtained using this calculation. ACO3 relative phosphorylation level refers to the level of phosphorylation relative to the WT control sample. Overview of the ACO3 peptides targeted in PRM and tSIM/PRM is presented in Supplemental Data Set 3.

### Gel electrophoresis and immunoblotting

Protein complex formation was assessed by separating soluble protein fractions corresponding to 5 µg of protein on CN PAGE with a 7.5%–12% (w/v) gradient of acrylamide as described in Järvi et al. (2011). The protein samples were loaded in 25BTH20G (25 mM BisTris/HCl [pH 7.0], 20% (w/v) glycerol) buffer supplemented with 0.05% (w/v) sodium deoxycholate. Preparation of 6% acrylamide, 50 µM Phos-tag gels, and protein separation on the gels was performed according to manufacturer’s instructions (www.wako-chem.co.jp/english/labchem). For analysis of protein abundance by immunoblotting, the protein samples were solubilized at 56°C for 15 min in Laemmli buffer and 30, 50, or 80 μg of protein was loaded for the detection of ACOs, SULPHOTRANSFERASE 12 (SOT12) and AOXs, respectively. For analysis of ACO and ACO3-YFP, proteins were separated by 10% SDS-PAGE, while 15% SDS-PAGE was used for the analysis of SOT12 and AOXs. Proteins were transferred onto a PVDF membrane and detected by α-ACO (Agrisera, Vännäs, Sweden; AS09 521), α-YFP (antibodies-online ABIN1545635), α-SOT12 (Agrisera AS16 3943), or α-AOX (Agrisera AS04 054) antibodies, using horseradish peroxidase‐linked secondary antibody and enhanced chemiluminescence (Amersham, GE Healthcare, Little Chalfont, UK) for detection.

### Enzymatic ACO assay

Protein from 4-week-old Arabidopsis rosettes was isolated by grinding the leaf material in 50 mM HEPES KOH pH 7.8, 2 mM MgCl_2_, 40 mM KCl, 1 mM EDTA, 0.1% (w/v) BSA, 1% (w/v) PVP 40,000 and 10% (v/v) glycerol supplemented with protease (Complete-Mini, Roche) and phosphatase (PhosSTOP, Roche) inhibitor cocktails. Protein was quantified with Protein Assay Dye Reagent (no. 5000006; Bio-Rad, Hercules, CA, USA). Total ACO activity was measured with Aconitase Assay Kit (no. MAK051; Millipore Sigma, Burlington, MA, USA) as nanomoles of isocitrate produced per mg of protein (mU mg^−1^ of protein) per minute following manufacturer’s instructions. A blank, corresponding to an assay in the absence of ACO substrate, was measured for every sample. Blank absorbance values were used for background correction.

### Phylogenetic analysis

*Arabidopsis thaliana* ACO3 (AT2G05710) orthologs were gathered from the comparative genomics databases Dicots PLAZA version 4.0, Moncots PLAZA version 4.5, Gymno PLAZA version 1.0 and Green Plants in pico-PLAZA version 3.0 (Van Bel et al., 2012) using the Integrative Orthology Viewer. Best-Hits-and-Inparalogs family genes were selected and used for the analysis. Protein sequences were aligned using the M-Coffee consensus alignment method available in the T-Coffee Multiple Sequence Alignment Server (Wallace et al., 2006; Di Tommaso et al., 2011) with default parameters. Alignments were filter-curated through the TCS algorithm (Chang et al., 2014). Curated M-Coffee alignments were imported into the bioinformatic suite Geneious Prime version 2020.1.2 (Biomaters Inc., Auckland, New Zealand) for the local generation of UPGMA consensus phylogenetic trees using Jukes-Cantor genetic distance model and 1,000 bootstraping repeats. Sequence logos were generated with Geneious Prime version 2020.1.2 (Biomaters Inc.).

### Statistical analysis

Statistical analysis was performed using the computing R environment (R Development Core Team, 2012) run in RStudio 1.2.5001 (Team Rs, 2015). Barplots and boxplots were generated with ggplot2 (Wickham, 2009).

## Accession numbers

Sequence data from this article can be found in the EMBL/GenBank data libraries under accession numbers ACO1 (AT4G35830), ACO2 (AT4G26970), ACO3 (AT2G05710), RBCL (ATCG00490), RBCS1A (AT1G67090), and GAPA (AT3G26650). The Skyline files containing refined PRM and tSIM/PRM data as well as the DDA data generated for constructing the spectral libraries for the targeted proteomics experiments, including RAW files, have been uploaded to Panorama Public and can be obtained from https://panoramaweb.org/iWNRyH.url or through the ProteomeXchange Consortium (PXD018881). DDA data has been uploaded in parallel to PRIDE and can be found at the ProteomeXchange Consortium (PXD024316).

## Supplemental data

The following materials are available in the online version of this article.

**Supplemental Figure S1.** ACO3localization in Arabidopsis leaf mesophyll cells.

**Supplemental Figure S2.** Characterization of *ACO3-YFP*, *ACO3S91A-YFP*, and *ACO3-OX* independent mutant lines.

**Supplemental Figure S3.** Relative abundance of proteins used to follow protein loading in the quantification of ACOs measured by PRM in the stress assay experiments.

**Supplemental Figure S4.** Adjustments in stress-inducible components in *rcd1* and *anac017*.

**Supplemental Figure S5.** Analysis of ACO phosphorylated forms in Phos-tag gels.

**Supplemental Figure S6.** Chlorophyll fluorescence kinetics in WT and *ACO3* complementation lines treated with control mock, MV, or MV + SHAM.

**Supplemental Figure S7.** Importance of ACO3 and its regulation by reversible phosphorylation at Ser91 in tolerance to AA-induced mitochondrial dysfunction.

**Supplemental Table S1.** List of primers used in this study.

**Supplemental Table S2.** List of proteins identified with high confidence in DDA mass spectrometry analysis of total protein extracts and ACO-containing gel bands.

**Supplemental Table S3.** List of high-confidence peptide groups identified for ACO1, ACO2, ACO3, RBCL, GAPA-1, and RBCS.

**Supplemental Table S4.** List of peptides targeted in PRM and tSIM/PRM analysis.

**Supplemental Data Set 1.** M-Coffee and TCS weight curated alignments of ACO3 orthologs.

**Supplemental Data Set 2.** Identification of the MDM in the *ACO3*gene promoter.

**Supplemental Data Set 3.** Overview of ACO3 PRM and tSIM/PRM peaks.

## Supplementary Material

kiab225_Supplementary_Data

## Notes

One-sentence summary**:** ACONITASE 3 contributes to mitochondrial dysfunction signaling and stress tolerance in Arabidopsis.

S.K., J.P., and M.R. conceived the original research plans; S.K., J.W., J.K., J.S., A.S., and A.T. supervised the experiments; J.P., M.R., M.A., S.A., G.D., R.G., A.S., and Z.W. performed the experiments; J.P and A.H designed the mass spectrometry experiments and analyzed the data; J.P., M.R., A.S., J.W., and S.K. conceived the project and wrote the article with contributions of all the authors; S.K. supervised and completed the writing. S.K. agrees to serve as the author responsible for contact and ensures communication.

The author responsible for distribution of materials integral to the findings presented in this article in accordance with the policy described in the Instructions for Authors (https://academic.oup.com/plphys/pages/general-instructions) is: Saijaliisa Kangasjärvi (saijaliisa.kangasjarvi@helsinki.fi).
