# Supplementary material for: ACONITASE 3 is part of theANAC017 transcription factor-dependent mitochondrial dysfunction response
Source: Plant Physiol. 2021 May 12;186(4):1859–77. doi: 10.1093/plphys/kiab225 (PMC8331168; doi:10.1093/plphys/kiab225)
Supplement: kiab225_Supplementary_Data [file kiab225_supplementary_data.zip › pp.01369.2020-s01.pdf]

Supplemental Figure S1

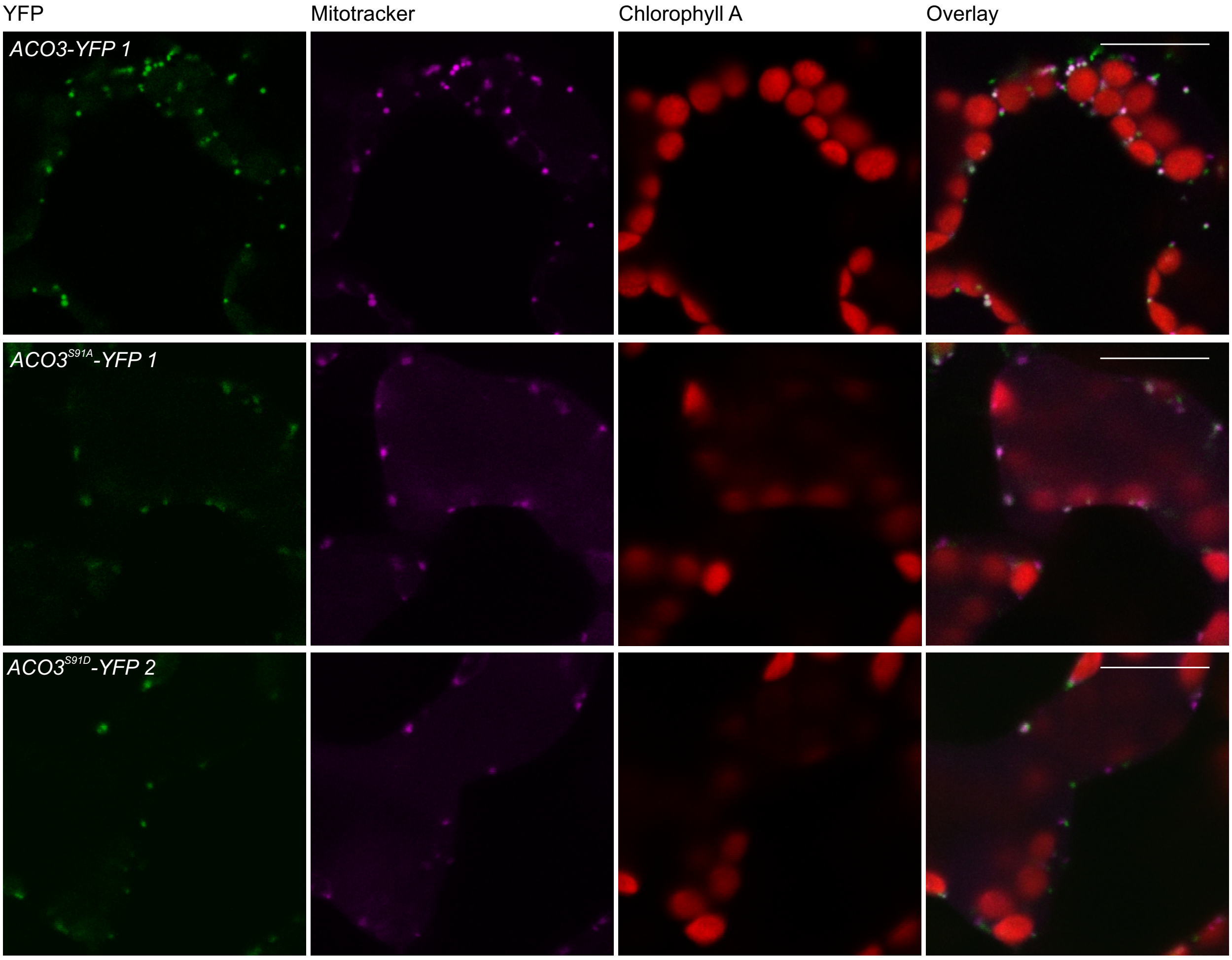

Supplemental Figure S1. ACONITASE 3 localization in Arabidopsis leaf mesophyll cells. Fluorescence confocal microscope images depicting ACO3-YFP localization in leaf mesophyll of the *aco3* lines expressing *pACO3::ACO3-YFP*, *pACO3::ACO3^{S91A}-YFP* or *pACO3::ACO3^{S91D}-YFP*. Scale bars correspond to 20 µm.

Supplemental Figure S2

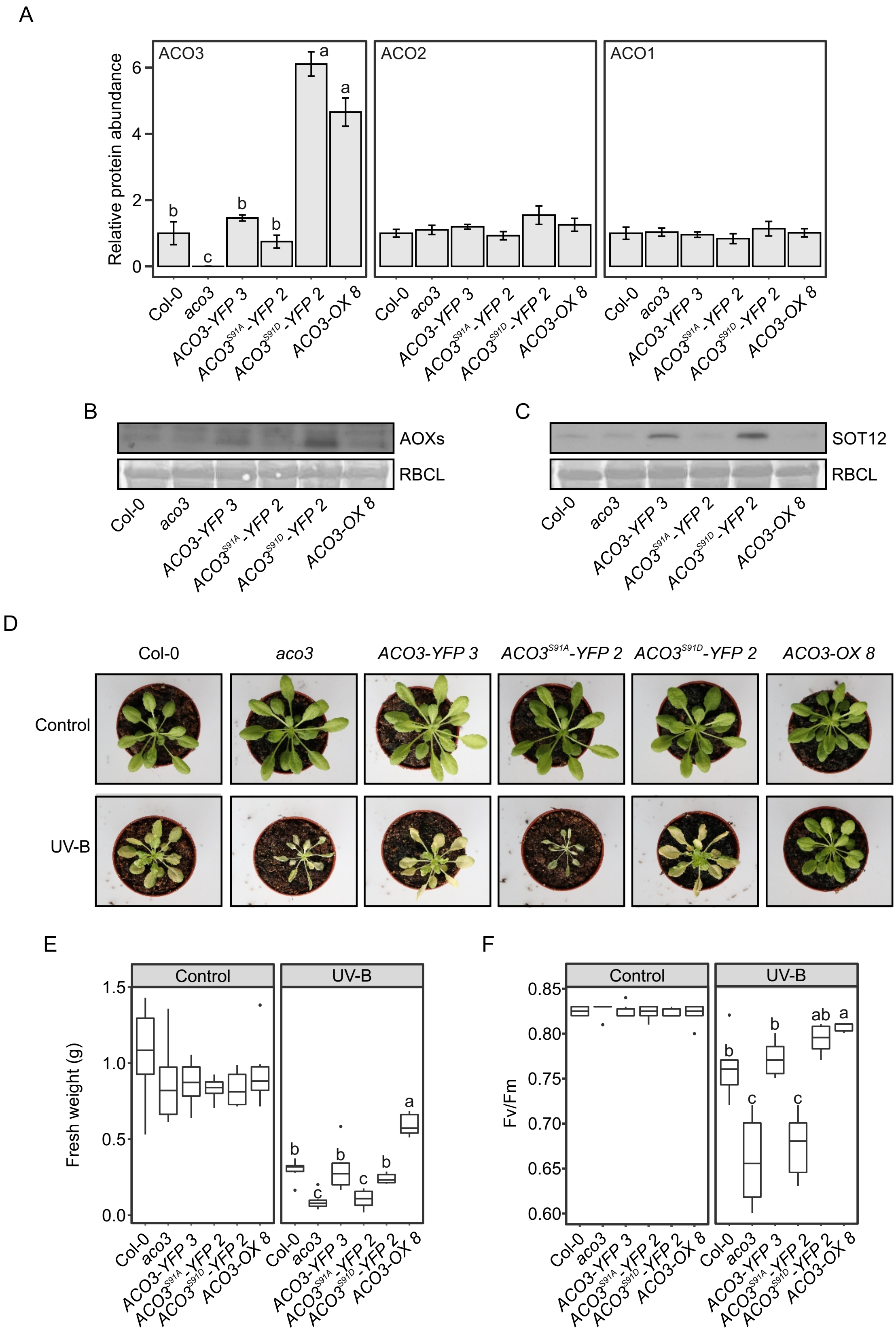

Supplemental Figure S2. Characterization of *ACO3-YFP*, *ACO3^{S91A}-YFP* and *ACO3-OX* independent mutant lines. A, Relative abundance of ACO1-3 isoforms and the relative total ACO protein abundance in WT (Col-0), *aco3, aco3* expressing *pACO3::ACO3-YFP* (*ACO3-YFP 3*), *pACO3::ACO3^{S91A}-YFP* (*ACO3^{S91A}-YFP 2*), *pACO3::ACO3^{S91D}-YFP* (*ACO3^{S91D}-YFP 2*) or *35S::ACO3* (*ACO3-OX 8*) as detected by parallel reaction monitoring (PRM). Values represent the mean of $n = 3 \pm$ SD. Letters indicate significant differences (One-way ANOVA with Tukey's HSD test, $p < 0.05$). B, Protein abundance of ALTERNATIVE OXIDASES (AOXs) in WT (Col-0), *aco3, aco3* expressing *pACO3::ACO3-YFP* (*ACO3-YFP 3*), *pACO3::ACO3^{S91A}-YFP* (*ACO3^{S91A}-YFP 2*), *pACO3::ACO3^{S91D}-YFP* (*ACO3^{S91D}-YFP 2*) or *35S::ACO3* (*ACO3-OX 8*) in control conditions as detected by immunoblotting. Coomassie staining of Rubisco large subunit (RBCL) is shown to demonstrate the equal loading of samples. C, Protein abundance of SULPHOTRANSFERASE 12 (SOT12) in WT (Col-0), *aco3, aco3* expressing *pACO3::ACO3-YFP* (*ACO3-YFP 3*), *pACO3::ACO3^{S91A}-YFP* (*ACO3^{S91A}-YFP 2*), *pACO3::ACO3^{S91D}-YFP* (*ACO3^{S91D}-YFP 2*) or *35S::ACO3* (*ACO3-OX 8*) in control conditions as detected by immunoblotting. Coomassie staining of Rubisco large subunit (RBCL) is shown to demonstrate the equal loading of samples. D, Phenotypes of WT (Col-0), *aco3*, *aco3* expressing *pACO3::ACO3-YFP* (*ACO3-YFP 3*), *pACO3::ACO3^{S91A}-YFP* (*ACO3^{S91A}-YFP 2*), *pACO3::ACO3^{S91D}-YFP* (*ACO3^{S91D}-YFP 2*) or *35S::ACO3* (*ACO3-OX 8*), 11 days after UV-B treatment (1.5 W m^{-2}, 45 minutes). The untreated control plants were kept under growth light. E, Fresh weight of the control and UV-B exposed plants 11 days after the treatment ($n = 6$). Letters indicate significant differences (Kruskal-Wallis test and Dunn-Bonferroni post-hoc test, $p < 0.05$). F, Maximum efficiency of PSII (Fv/Fm) of the control and UV-B exposed plants 11 days after the treatment ($n = 6$). Letters indicate significant differences (Kruskal-Wallis test and Fisher´s least significant difference post-hoc test with Bonferroni adjustment , $p < 0.05$). E and F, The box plots show the median (central line), the lower and upper quartiles (box), the minimum and maximum values within 1.5X the interquartile range (whiskers) and the outliers (points).

Supplemental Figure S3

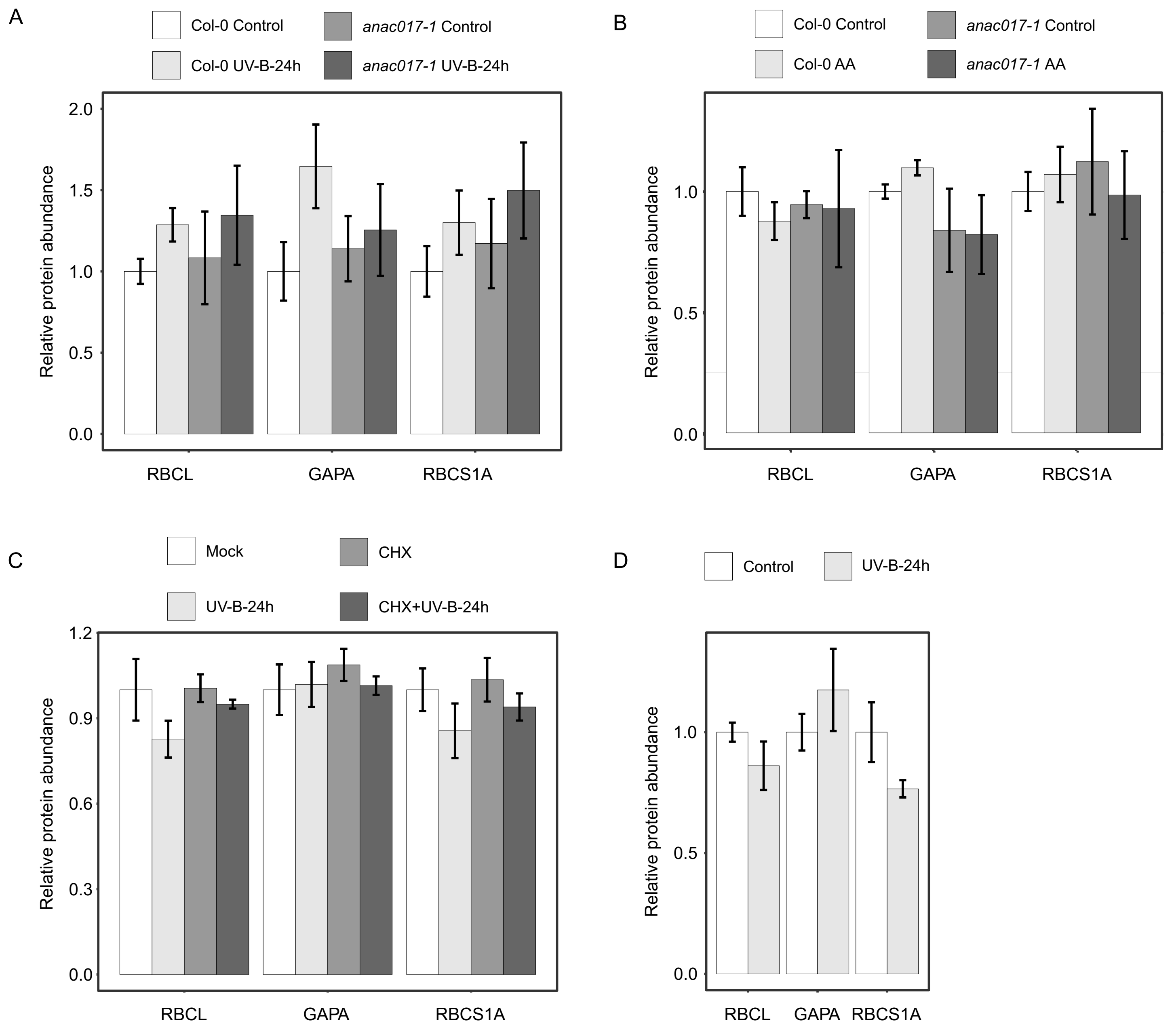

Supplemental Figure S3. Relative abundance of proteins used to follow protein loading in the quantification of aconitases measured by Parallel Reaction Monitoring (PRM) in the stress assay experiments. A, Relative protein abundance of RUBISCO LARGE SUBUNIT (RBCL), RUBISCO SMALL SUBUNIT 1A (RBCS1A) and GLYCERALDEHYDE 3-PHOSPHATE DEHYDROGENASE A SUBUNIT (GAPA) in control and UV-B-treated WT (Col-0) and *anac017-1* plants as measured by parallel reaction monitoring (PRM). B, Relative protein abundance of RUBISCO LARGE SUBUNIT (RBCL), RUBISCO SMALL SUBUNIT 1A (RBCS1A) and GLYCERALDEHYDE 3-PHOSPHATE DEHYDROGENASE A SUBUNIT (GAPA) in control and AA-treated WT (Col-0) and *anac017-1* plants measured by parallel reaction monitoring (PRM). C, Relative protein abundance of RUBISCO LARGE SUBUNIT (RBCL), RUBISCO SMALL SUBUNIT 1A (RBCS1A) and GLYCERALDEHYDE 3-PHOSPHATE DEHYDROGENASE A SUBUNIT (GAPA) in WT (Col-0) plants in in control conditions (Mock), 24h after treatment with UV-B (1.5 W m^{-2}, 45 minutes), after treatment with 25 µM cycloheximide (CHX), and after CHX treatment followed by UV-B treatment as measured by parallel reaction monitoring (PRM). D, Relative protein abundance of RUBISCO LARGE SUBUNIT (RBCL), RUBISCO SMALL SUBUNIT 1A (RBCS1A) and GLYCERALDEHYDE 3-PHOSPHATE DEHYDROGENASE A SUBUNIT (GAPA) in control and UV-B-treated *35S::ACO3* over-expression line *ACO3-OX 3* as measured by parallel reaction monitoring (PRM). A-D, Error bars represent SD (n = 3; One-way ANOVA).

Supplemental Figure S4

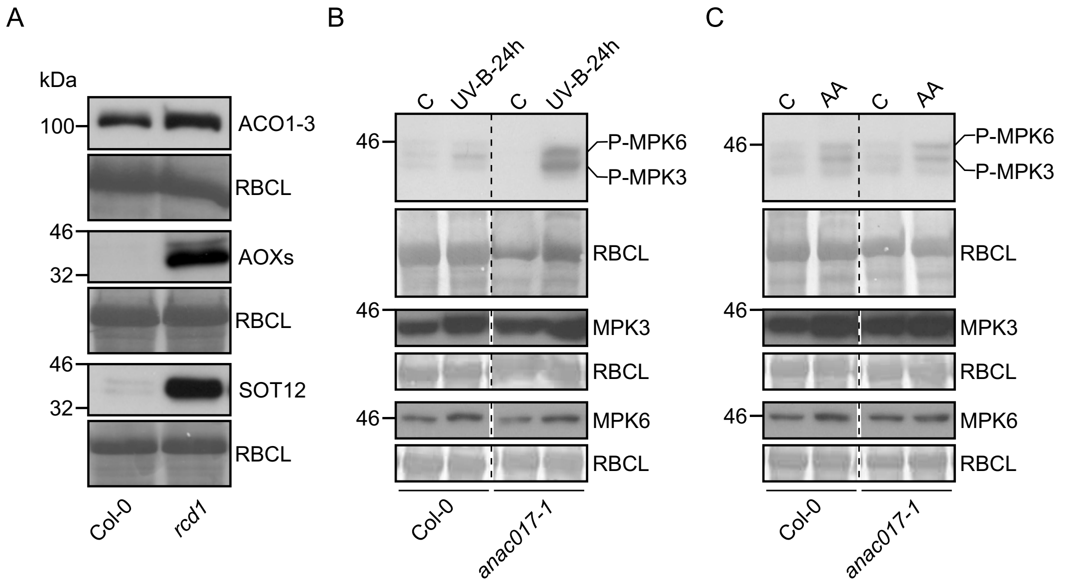

Supplemental Figure S4. Adjustments in stress-inducible components in *rcd1* and *anac017*. A, Abundance of ACONITASE1-3, and of the MDR marker proteins ALTERNATIVE OXIDASES (AOXs) and SULPHOTRANSFERASE 12 (SOT12) in WT and r*cd1* mutant plants. Coomassie staining of RUBISCO LARGE SUBUNIT (RBCL) is shown to demonstrate equal loading of samples. B, UV-B-induced phosphorylation of MPK3 and MPK6 in Col-0 and *anac017-1* 24 hours after the treatment as detected by immunoblotting using an anti-pTpY antibody against activated MPK3 and MPK6, and total MPK3 and MPK6 abundances. Coomassie stainings of RUBISCO LARGE SUBUNIT (RBCL) are shown to demonstrate equal loading of samples. C, AA-induced phosphorylation of MPK3 and MPK6 in Col-0 and *anac017-1* 10 hours after the treatment as detected by immunoblotting using an anti-pTpY antibody against activated MPK3 and MPK6, and total MPK3 and MPK6 abundances. Coomassie stainings of RUBISCO LARGE SUBUNIT (RBCL) are shown to demonstrate equal loading of samples.

Supplemental Figure S5

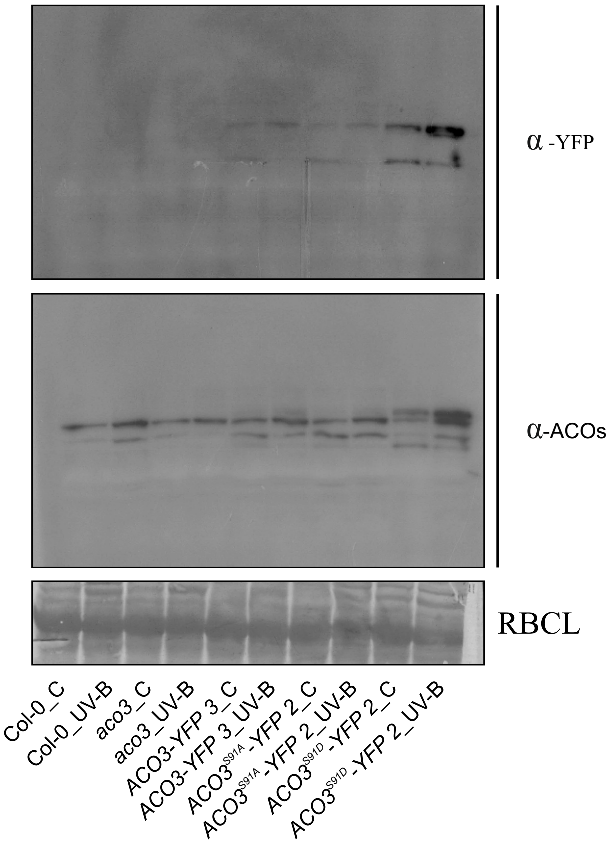

Supplemental Figure S5. Analysis of ACO phosphorylated forms in Phos-tag gels. ACO immunoblots after separation of total leaf protein extracts on Phos-tag gels and detection by anti-YFP and anti-ACO antibodies.

Supplemental Figure S6

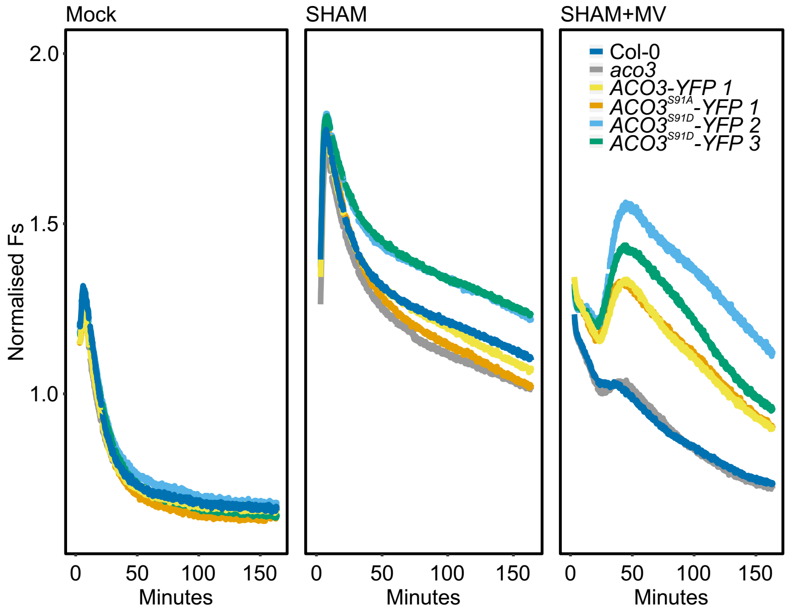

Supplemental Figure S6. Chlorophyll fluorescence kinetics in wild type and *ACONITASE 3* complementation lines treated with control mock, methyl viologen (MV), or MV + salicylhydroxamic acid (SHAM). Kinetics of chlorophyll fluorescence (Fs) in WT (Col-0), *aco3*, and *aco3* complemented by *pACO3::ACO3-YFP* (*ACO3-YFP 1*), *pACO3::ACO3*^{S91A}*-YFP* (*ACO3*^{S91A}*-YFP 1*) or *pACO3::ACO3*^{S91D}*-YFP* (*ACO3*^{S91D}*-YFP 2* and *ACO3*^{S91D}*-YFP 3*) after control treatment and after treatment with SHAM alone or with 2 mM SHAM and 1 µM MV. Chlorophyll fluorescence was measured using an imaging PAM under actinic illumination of 80 µmol photons m^{-2} sec^{-1}.

Supplemental Figure S7

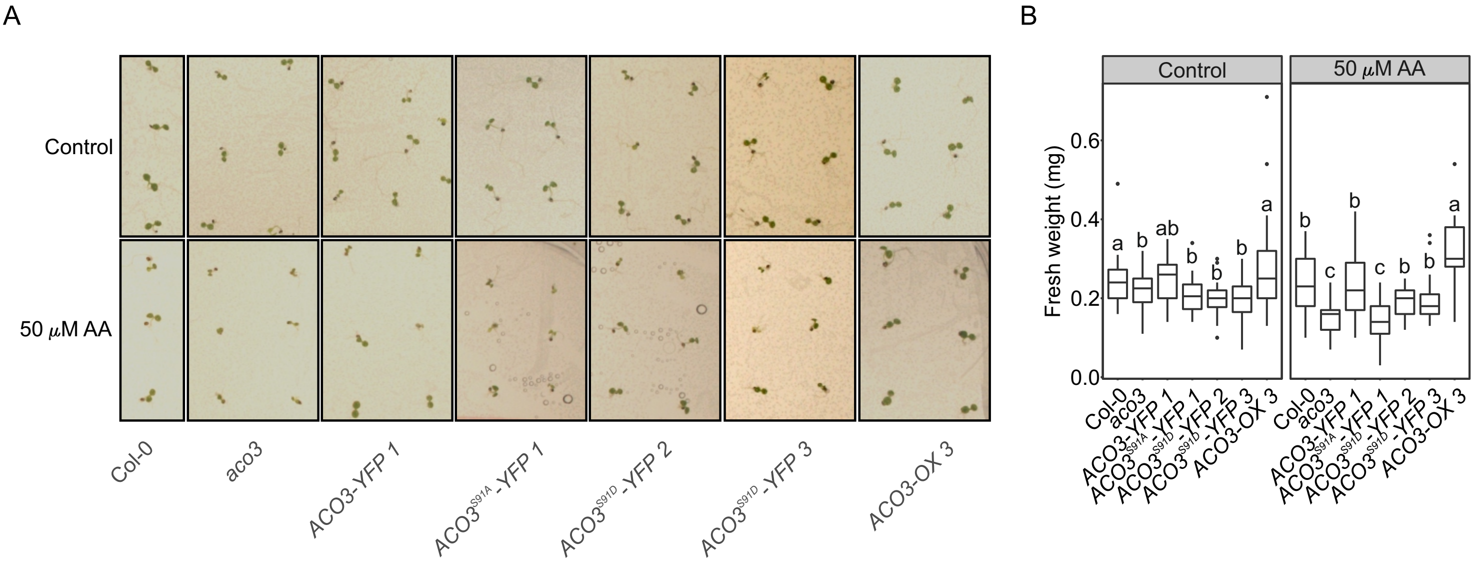

Supplemental Figure S7. Importance of ACONITASE 3 and its regulation by reversible phosphorylation at Ser91 in tolerance to antimycin A-induced mitochondrial dysfunction. A, Photographs and (B) fresh weight of one-week-old seedlings of WT (Col-0), *aco3*, *aco3* expressing *pACO3::ACO3-YFP* (*ACO3-YFP 1*), *pACO3::ACO3*^{S91A}*-YFP* (*ACO3*^{S91A}*-YFP 1*), *pACO3::ACO3*^{S91D}*-YFP* (*ACO3*^{S91D}*-YFP 2* and *ACO3*^{S91D}*-YFP 3*) or *35S::ACO3* (*ACO3-OX 3*), germinated on ½ MS medium with or without 50 μM antimycin A (AA; $n \geq 19$). The box plots show the median (central line), the lower and upper quartiles (box), the minimum and maximum values within 1.5X the interquartile range (whiskers) and the outliers (points). Letters indicate significant differences (Kruskal-Wallis test and Fisher´s least significant difference post-hoc test with Bonferroni adjustment, $p < 0.05$).
