# Supplementary material for: ACONITASE 3 is part of theANAC017 transcription factor-dependent mitochondrial dysfunction response
Source: Plant Physiol. 2021 May 12;186(4):1859–77. doi: 10.1093/plphys/kiab225 (PMC8331168; doi:10.1093/plphys/kiab225)
Supplement: kiab225_Supplementary_Data [file kiab225_supplementary_data.zip › pp.01369.2020-s03.pdf]

Supplemental Dataset S2. Identification of the mitochondrial dysfunction motif in the *ACONITASE 3* gene promoter.

The mitochondrial dysfunction motif (MDM) identified by De Clercq et al. (2013) was used to locate putative ANAC017 binding sites in the *ACO3* promoter. The 40 MDM sequences that form the 24 mitochondrial dysfunction stimulon (MDS) genes in De Clercq et al. (2013) were used to calculate a position weight matrix (PWM) (A) and a sequence logo (B) for the MDM using RSAT Convert Matrix tool (Nguyen et al. 2018, http://embnet.ccg.unam.mx/rsat/convert-matrix_form.cgi). The MDM PWM was searched against the 2631 bp *ACO3* promoter area upstream of the translational start codon using RSAT Matrix Scan (Turatsinze et al. 2008, http://embnet.ccg.unam.mx/rsat/matrix-scan-quick_form.cgi) (C). The identified putative MDM motives in *ACO3* promoter are highlighted in grey and the highly conserved areas of the MDM are underlined (D). The 5' UTR region of ACO3 is indicated with lower case letters.

References

De Clercq I, Vermeirssen V, Van Aken O, Vandepoele K, Murcha MW, Law SR, Inze A, Ng S, Ivanova A, Rombaut D, *et al.* 2013. The membrane-bound NAC transcription factor ANAC013 functions in mitochondrial retrograde regulation of the oxidative stress response in Arabidopsis. *Plant Cell* **25**: 3472–3490.

Nguyen NTT, Contreras-Moreira B, Castro-Mondragon JA, Santana-Garcia W, Ossio R, Robles-Espinoza CD, Bahin M, Collombet S, Vincens P, Thieffry D, van Helden J, Medina-Rivera A, Thomas-Chollier M. 2018. RSAT 2018: regulatory sequence analysis tools 20th anniversary. *Nucleic Acids Research* **2**: W209-W214.

Turatsinze JV, Thomas-Chollier M, Defrance M, van Helden J. 2008. Using RSAT to scan genome sequences for transcription factor binding sites and cis-regulatory modules. *Nature Protocols* **3**: 1578-1588.

A

A	[9	6	13	0	0	0	1	7	20	6	32	12	4	40	31	3	12	13	17]
C	[5	3	10	37	0	0	0	6	8	7	2	9	32	0	9	2	9	4	5]
G	[7	5	9	0	0	0	39	22	8	25	5	13	4	0	0	35	4	3	9]
T	[19	26	8	3	40	40	0	5	4	2	1	6	0	0	0	0	15	20	9]

B

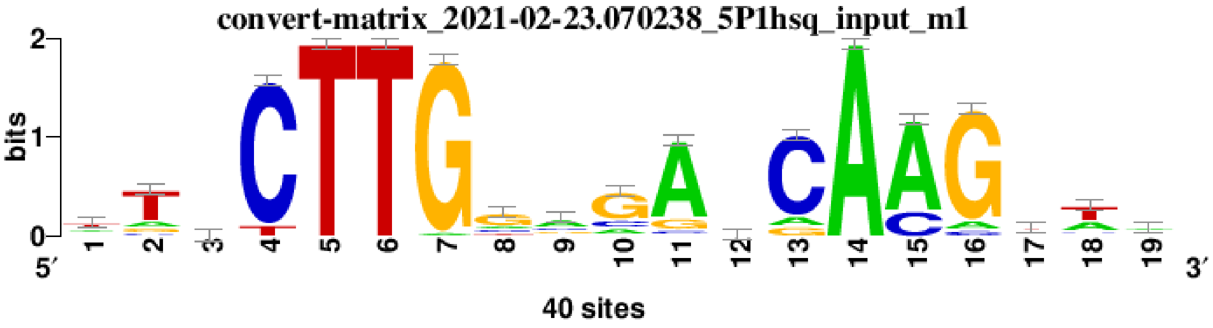

C

```
matrix-scan  -v 1 -quick -matrix_format tab -m $RSAT/public_html/tmp/apache/2021/02/24/matrix-scan_2021-02-24.021715_a38MiT.matrix -pseudo 1 -decimals 1 -2str -origin end -bginput -markov 1 -bg_pseudo 0.01 -return limits -return pval -uth pval 0.001 -i $RSAT/public_html/tmp/apache/2021/02/24/tmp_sequence_2021-02-24.021715_1eU4Cz.fasta -seq_format fasta -n score
 Quick counting mode
 Input files
         input     $RSAT/public_html/tmp/apache/2021/02/24/tmp_sequence_2021-02-24.021715_1eU4Cz.fasta
 Matrix files
         matrix 1  $RSAT/public_html/tmp/apache/2021/02/24/matrix-scan_2021-02-24.021715_a38MiT.matrix
 Sequence format           fasta
 Pseudo counts             1
 Background model
         Method            input
         Markov order      1
         Strand            undef
         Background pseudo-frequency  0.01
         Residue probabilities
                   a       0.37621
                   c       0.11898
                   g       0.13321
                   t       0.37159
 Thresholds        lower   upper
         pval      NA      0.001
 Output columns
         1         seq_id
         2         ft_type
         3         ft_name
         4         strand
         5         start
         6         end
         7         sequence
         8         weight
```

**seq_id**	**ft_type**	**ft_name**	**strand**	**start**	**end**	**sequence**	**weight**	**Pval**	**ln_Pval**	**sig**
At2g05710	limit	START_END	D	-2631	-1	.	0	0	0	0
At2g05710	site	matrix-scan_2021-02-24	R	-2353	-2335	CTTCCTGCCAAACCCGTCC	2.7	6.5e-04	-7.339	3.187
At2g05710	site	matrix-scan_2021-02-24	D	-2350	-2332	CGGGTTTGGCAGGAAGCTA	2.7	6.5e-04	-7.339	3.187
At2g05710	site	matrix-scan_2021-02-24	R	-1332	-1314	TTGTTTGCTACCAAACCTC	2.3	8.1e-04	-7.118	3.092
At2g05710	site	matrix-scan_2021-02-24	D	-1090	-1072	TATCTTGTCGGAAACATGT	2.9	6.2e-04	-7.386	3.208
At2g05710	site	matrix-scan_2021-02-24	D	-1015	-997	TTTTTTGGAAAAAACATTG	2.5	7.7e-04	-7.169	3.114
At2g05710	site	matrix-scan_2021-02-24	R	-405	-387	CTGTTTGAAAAAGACAACG	2.2	8.5e-04	-7.070	3.071
At2g05710	site	matrix-scan_2021-02-24	R	-368	-350	TTACTTAAAAATCAAATAA	2.4	7.7e-04	-7.169	3.114
At2g05710	site	matrix-scan_2021-02-24	D	-225	-207	TCCCTTGCGAGAGAGGGAA	4.7	2.1e-04	-8.468	3.678
At2g05710	site	matrix-scan_2021-02-24	R	-173	-155	GCGTGTGGAGACGAAACGA	2.6	7.3e-04	-7.222	3.137

```
 Matrices
         matrix    name                       ncol      nrow      pseudo    Wmin      Wmax      Wrange
         1         matrix-scan_2021-02-24     19        4         0         -40.500   19.400    59.900    a:0.376 c:0.119 g:0.133 t:0.372
 Number of sequences scanned 1
 Sum of sequence lengths     2631
```

```
 N residues                  0
 Matches per matrix
         matrix    name                         matches    scored
         1         matrix-scan_2021-02-24       9          0
                   TOTAL              9         0
 Host name         sinik
 Job started       2021-02-24.021716
 Job done 2021-02-24.021719
 Seconds  1.72
         user      1.72
         system    0.03
         cuser     1.98
;        csystem   0.07
```

D

```
ACCTTTCCAAAATCTCTCTGCATTAACTATACGATTAATTACTAAAATAAAATTTCCAAAATATTTAATATCATTTAAAT
ACTACAAAATTATCATTTTTGATATTGCTTTTTTTTTATGACTATAACAATTCGATTATAAGCAGCAAACCGTAAAGATA
TTTGATAGCAATTAATTACTACAAAATTATAAAATATTTAGACAATGATTCATAAACATATCATAAATAAGATCAACATT
AATAAAATAAATAGTTTTTTTTACGAGACGGGTTGGCGGGACGGGTTTGGCAGGAAGCTACTTAATAACAATTGTAAACT
ATAAAATAAAAATATTTTATAGATAGATAAAATTTGTAAACTTTTATATATACTAACTTTAAGGAAATAAATTGTCTCCG
CGGTATACCGCGGGTTAAAATCTAGTTAATTAGTGTTATCGACTCATGATTAAATATATAGAATTGATATAAACTGAACA
AGATTTCAAAAGTATTCATATGAAAAGAATTAAAAAAAAAAGCATAAATTAAAGCAGTATTTAAGTCAATACTTTTAGAT
CTTCTCTCATTGCATATATCAATATAAGAGAAATGATCGTGTATTAAATTATTTGGTAAGTCTAACGTTTTAAAGGTTTA
TTATTTGAAAAAACTTGAGTGTCAATGTTATCAAAGACTCAAAATAAAATTACTAAACTAATAAGAGGTTAGGTAACGAA
CAAGTACAACACAAAAAAATTAGTAAAATAAAAAGAGTATAATAAAAGTAGTGCTTAATTTATTAACATAATTTTTTATT
TTATTTTTATCTTGAAAAAATCAATTAAGAGTAGGATTGATGATACATTTAATTTGCTTATTTAATATGATTAATATTTT
AATTTAAAAAATATAGAATGACACATGTCATTTTTTTGTGTGATGATGTGTCATTCATATGGAAAGAGTTGGCCAACTTT
CATATATATGATTAGATTTCTAAATGATCATTGCCGTAATATTAGTAAAGATGTGTCCAGTGTTTTAGCAATTTTTACCA
AACTAATTTTGATTTACATCTTTAATTAAATATTTCATTTATTAATAATTAATGTCAATAGCATGACATTGTAAATAAGT
TCAAGTACAAGATTTATTTGTTAAAGTAGCTGAAAAATGTATATGTAGATTTTAGATCTCTAAATAAGATTAACATAAGA
AATCTGTATCGGTCTAACTAATGATGAATCGCGAAGGACAACGTTAGAAAACTAATGACGAAAATATGTTCATGAGAATA
ATATATACTAAACTAATAAGAGGTTTGGTAGCAAACAACTACAACACAAAAAAAAGTCATTATCTAGTAGTGATTATTGA
TAATAAAAATTTAATAAAATAAAGAGTATAATGAAAGTAGTGCTTAATTTATTAACATAATTTTTTTTTATTTTATTTTG
AATCAATTAAGAATAGGATTAATCATACATTTAATTTTAGAGTATTTATAATAAATGACTATAGATTTGAAAAGAATGTA
CAAACTAACCTTGAGTCACAGTATCTTGTCGGAAACATGTTGGAGAACACTTGTTATTTTCTCTTATTACATAGTATTTA
TAAAAAGCACATGCTATTTTTTGGAAAAAACATTGTATTCAGTTTAATATATCTTTTTATCATGATATTTTGTAGTAATA
CACGTTTTTTATTGAGATCACACGTGATTTCTTTAAAAATATTCTTATTTTTTAGTAATACATGGTTTTTATTAAAATCA
CATATAATTTTTGTTTTAAGATTCTGTTCATGTAAAAGAATTTCATAATAATGTTTTAAAAAAAAAAATTCATAACGTAT
TATGAAATAGCATGTTTTTAAACTAAATATAACATGTGAAATTTTAATGTTACATGATATTTTGAAAAATTACACGGATG
TGTTTCGTTTACTTTTCATCTTACAACAAATAAAGGATAGCTTTATTATTTCCTTTCTTCCGACAACGTGGGTCCATGGT
TATTTTGTAACAAAAAACTTTATCTCTGCTAGTTTCTTAATTTGTATGTTTTATTTGGTTATCTACTAATATTACCTTTA
ATTTTATTATTTTTTAAATTTAAAAATATAGAATGATATATGTCAAACTTTTGTGTGATGCTTTTGTGTGATGGTGTGTC
ATTCATATGGAGAGTGTTGACCAATTTTCATATATATATGATTATATATTAAGAAACTATATATTACGTTGTCTTTTTCA
AACAGGTAAAAATGTTATGAGATTTATTTGATTTTTAAGTAATTATTTAAATTAAAATAAATAAAAATATATATTATTTT
AATAATAGGGGCTAAATAGAAATTATGTGGAGGAGAGGGGCTGCGTAGATTAAAATCCAAAAAATACCGTAAACAAAAAA
AAATATTCCCTTGCGAGAGAGGGAAGATTGACTTATTGGcacgcagccctaggcattttcgtttcgtctccacacgcctc
tctctctctctcgcttcttcaatcattcatcactccgacgtcagattcaaaatcctccatgatcgatcccaattgatcaa
tcaataaccttatcttcttcatcttctctgtcagtatctgaatctgcatctggaagcgtgatttcatcatc
```
