# Supplementary material for: ACONITASE 3 is part of theANAC017 transcription factor-dependent mitochondrial dysfunction response
Source: Plant Physiol. 2021 May 12;186(4):1859–77. doi: 10.1093/plphys/kiab225 (PMC8331168; doi:10.1093/plphys/kiab225)
Supplement: kiab225_Supplementary_Data [file kiab225_supplementary_data.zip › pp.01369.2020-s04.pdf]

Supplemental Dataset S3. Overview of ACONITASE 3 PRM and tSIM/PRM peaks.

Representative ion peaks corresponding to the ACO3 peptides targeted in PRM (A) and tSIM/PRM (B, C, D) analysis. A WT (Col-0) sample is shown in comparison to an *aco3* sample.

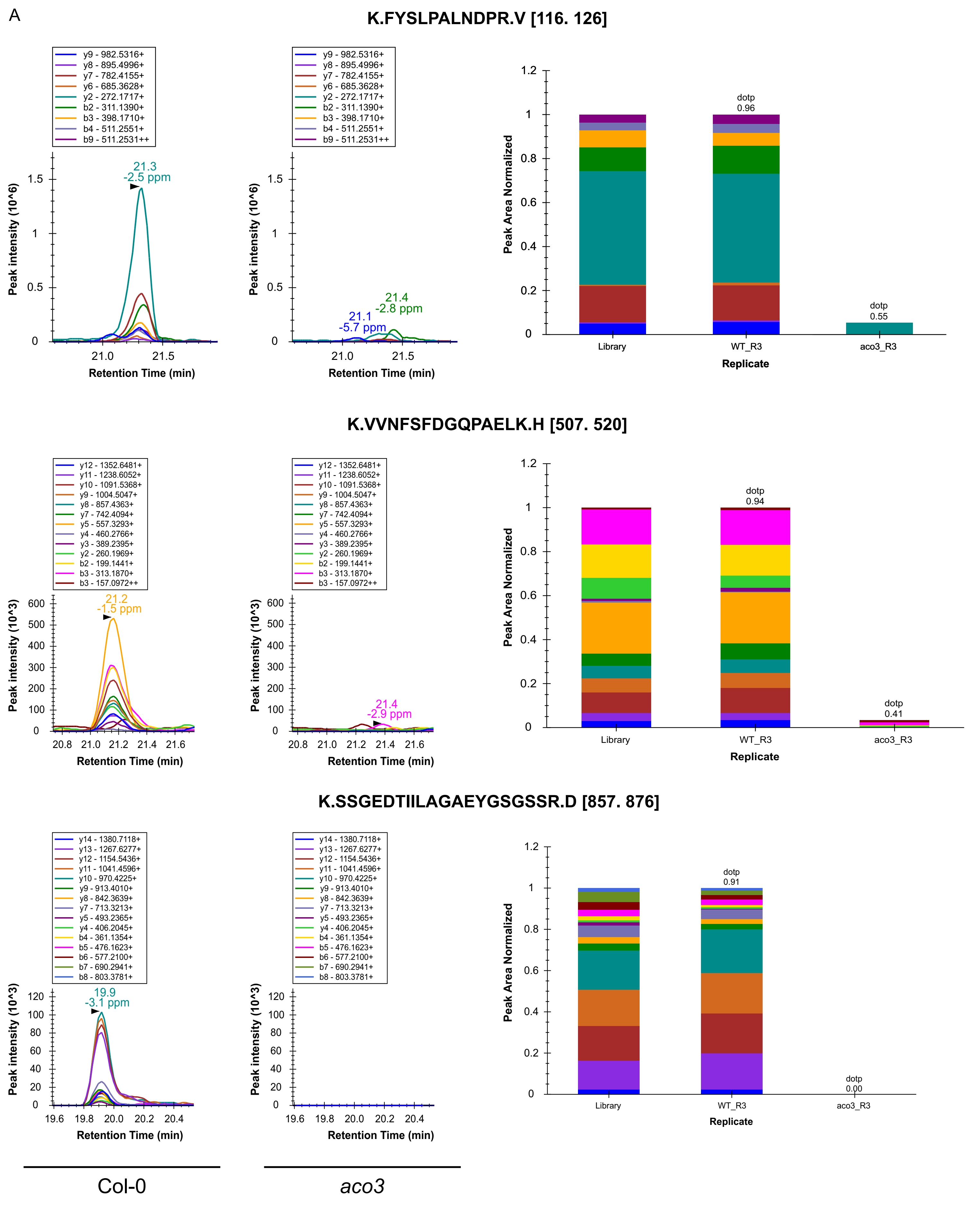

B

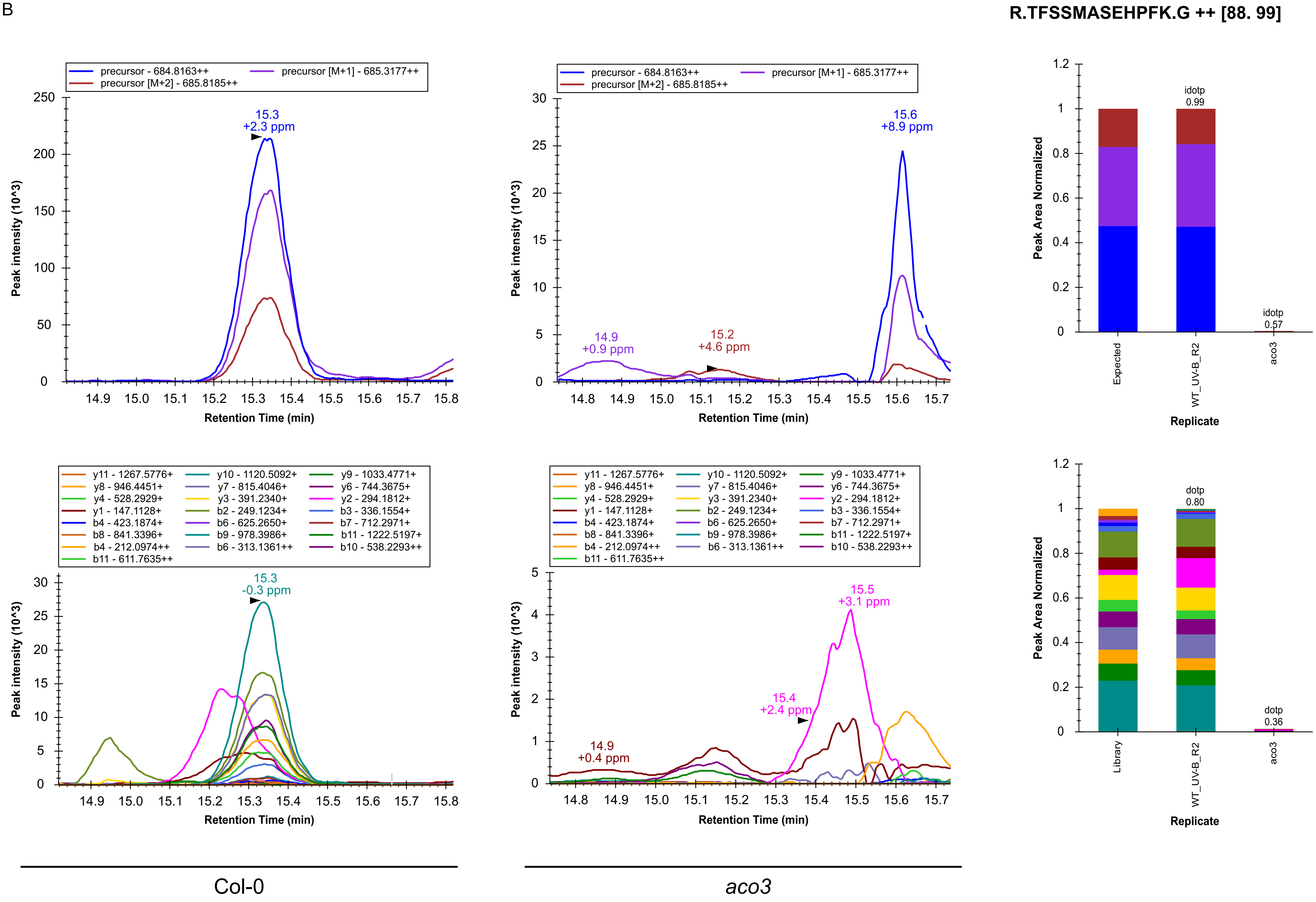
R.TFSSMASEHPFK.G ++ [88. 99]
precursor - 684.8163++
precursor [M+1] - 685.3177++
precursor [M+2] - 685.8185++
15.3
+2.3 ppm
Peak intensity (10^3)
Retention Time (min)
15.6
+8.9 ppm
14.9
+0.9 ppm
15.2
+4.6 ppm
Peak Area Normalized
idotp
0.99
idotp
0.57
Expected
WT_UV-B_R2
aco3
Replicate
y11 - 1267.5776+
y10 - 1120.5092+
y9 - 1033.4771+
y8 - 946.4451+
y7 - 815.4046+
y6 - 744.3675+
y4 - 528.2929+
y3 - 391.2340+
y2 - 294.1812+
y1 - 147.1128+
b2 - 249.1234+
b3 - 336.1554+
b4 - 423.1874+
b6 - 625.2650+
b7 - 712.2971+
b8 - 841.3396+
b9 - 978.3986+
b11 - 1222.5197+
b4 - 212.0974++
b6 - 313.1361++
b10 - 538.2293++
b11 - 611.7635++
15.3
-0.3 ppm
15.5
+3.1 ppm
15.4
+2.4 ppm
14.9
+0.4 ppm
dotp
0.80
dotp
0.36
Library
Col-0
aco3

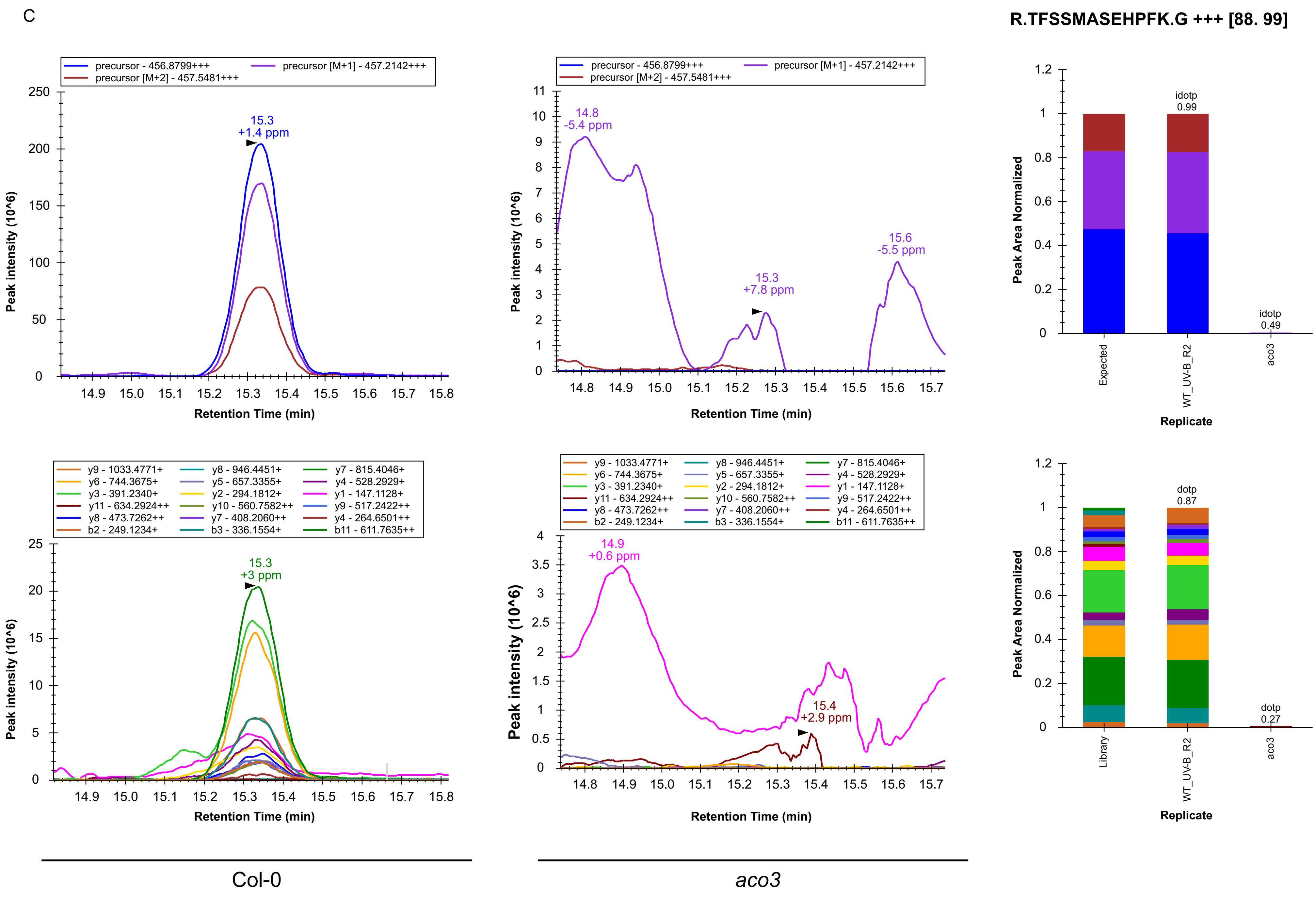
C
R.TFSSMASEHPFK.G +++ [88. 99]
precursor - 456.8799+++
precursor [M+1] - 457.2142+++
precursor [M+2] - 457.5481+++
15.3
+1.4 ppm
14.8
-5.4 ppm
15.3
+7.8 ppm
15.6
-5.5 ppm
Peak intensity (10^6)
Retention Time (min)
Peak Area Normalized
idotp
0.99
idotp
0.49
Expected
WT_UV-B_R2
aco3
Replicate
y9 - 1033.4771+
y8 - 946.4451+
y7 - 815.4046+
y6 - 744.3675+
y5 - 657.3355+
y4 - 528.2929+
y3 - 391.2340+
y2 - 294.1812+
y1 - 147.1128+
y11 - 634.2924++
y10 - 560.7582++
y9 - 517.2422++
y8 - 473.7262++
y7 - 408.2060++
y4 - 264.6501++
b2 - 249.1234+
b3 - 336.1554+
b11 - 611.7635++
15.3
+3 ppm
14.9
+0.6 ppm
15.4
+2.9 ppm
dotp
0.87
dotp
0.27
Library
Col-0
aco3

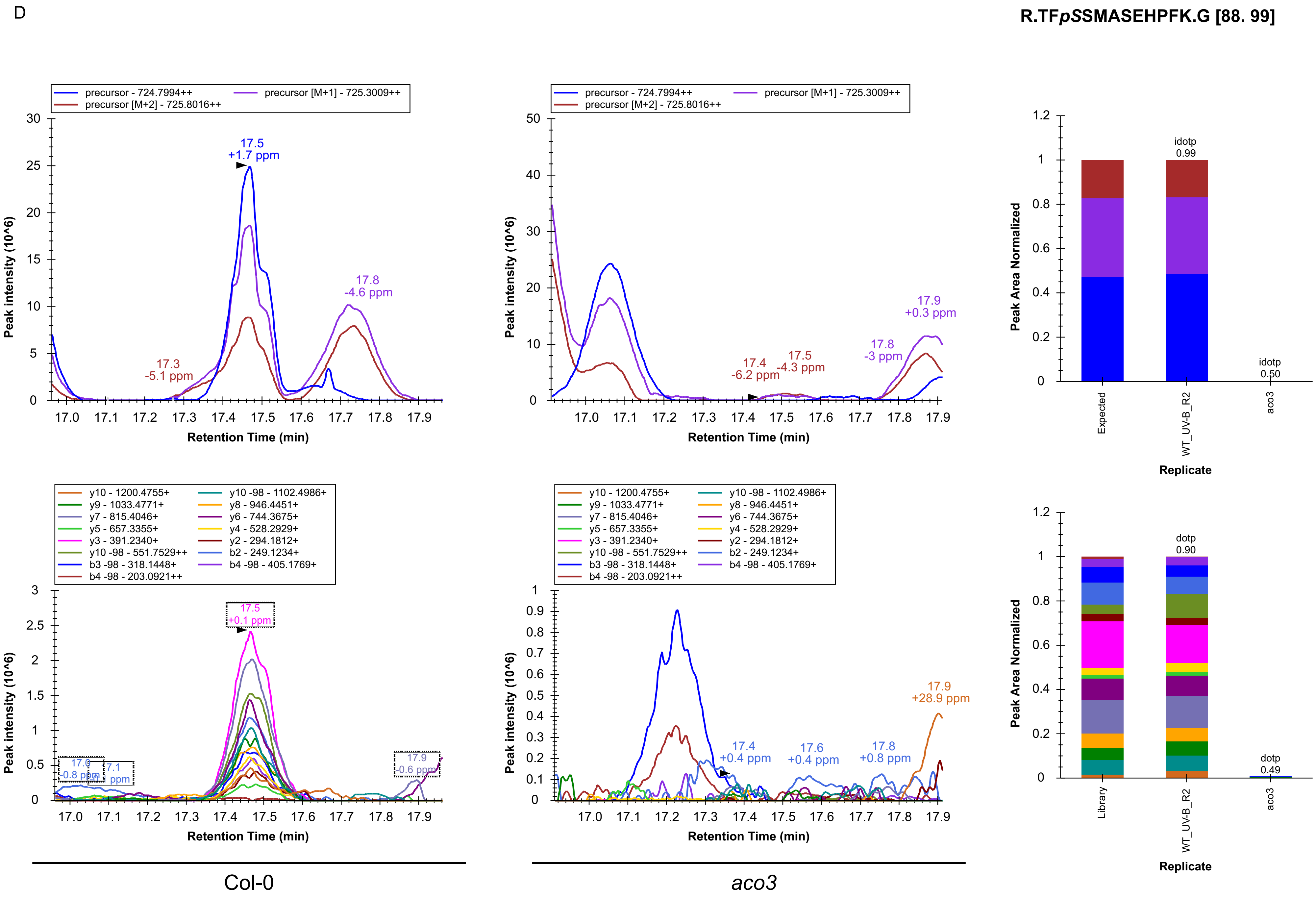
D
R.TFpSSMASEHPFK.G [88. 99]
precursor - 724.7994++
precursor [M+1] - 725.3009++
precursor [M+2] - 725.8016++
17.5
+1.7 ppm
17.8
-4.6 ppm
17.3
-5.1 ppm
17.9
+0.3 ppm
17.8
-3 ppm
17.4
-6.2 ppm
17.5
-4.3 ppm
Peak intensity (10^6)
Retention Time (min)
Peak Area Normalized
idotp
0.99
idotp
0.50
Expected
WT_UV-B_R2
aco3
Replicate
y10 - 1200.4755+
y10 -98 - 1102.4986+
y9 - 1033.4771+
y8 - 946.4451+
y7 - 815.4046+
y6 - 744.3675+
y5 - 657.3355+
y4 - 528.2929+
y3 - 391.2340+
y2 - 294.1812+
y10 -98 - 551.7529++
b2 - 249.1234+
b3 -98 - 318.1448+
b4 -98 - 405.1769+
b4 -98 - 203.0921++
17.5
+0.1 ppm
17.9
-0.6 ppm
17.9
+28.9 ppm
17.4
+0.4 ppm
17.6
+0.4 ppm
17.8
+0.8 ppm
dotp
0.90
dotp
0.49
Library
Col-0
aco3
